# The Rotterdam Study: 2018 update on objectives, design and main results

**DOI:** 10.1007/s10654-017-0321-4

**Published:** 2017-10-24

**Authors:** M. Arfan Ikram, Guy G. O. Brusselle, Sarwa Darwish Murad, Cornelia M. van Duijn, Oscar H. Franco, André Goedegebure, Caroline C. W. Klaver, Tamar E. C. Nijsten, Robin P. Peeters, Bruno H. Stricker, Henning Tiemeier, André G. Uitterlinden, Meike W. Vernooij, Albert Hofman

**Affiliations:** 1 Department of Epidemiology, Erasmus Medical Center, PO Box 2040, 3000 CA Rotterdam, The Netherlands; 2 Department of Internal Medicine, Erasmus Medical Center, Rotterdam, The Netherlands; 3 Department of Neurology, Erasmus Medical Center, Rotterdam, The Netherlands; 4 Department of Psychiatry, Erasmus Medical Center, Rotterdam, The Netherlands; 5 Department of Cardiology, Erasmus Medical Center, Rotterdam, The Netherlands; 6 Department of Radiology and Nuclear Medicine, Erasmus Medical Center, Rotterdam, The Netherlands; 7 Department of Ophthalmology, Erasmus Medical Center, Rotterdam, The Netherlands; 8 Department of Gastro-Enterology, Erasmus Medical Center, Rotterdam, The Netherlands; 9 Department of Otolaryngology, Erasmus Medical Center, Rotterdam, The Netherlands; 10 Department of Dermatology, Erasmus Medical Center, Rotterdam, The Netherlands; 11 Department of Respiratory Medicine, Erasmus Medical Center, Rotterdam, The Netherlands; 12 Department of Respiratory Medicine, Ghent University Hospital, Ghent, Belgium; 13 Department of Epidemiology, Harvard T. H. Chan School of Public Health, Boston, MA USA

**Keywords:** Biomarkers, Cardiovascular diseases, Cohort study, Dermatological diseases, Endocrine diseases, Epidemiologic methods, Genetic epidemiology, Liver diseases, Neurological diseases, Oncology, Ophthalmic diseases, Otolaryngological diseases, Pharmacoepidemiology, Renal diseases, Psychiatric diseases, Respiratory diseases

## Abstract

The Rotterdam Study is a prospective cohort study ongoing since 1990 in the city of Rotterdam in The Netherlands. The study targets cardiovascular, endocrine, hepatic, neurological, ophthalmic, psychiatric, dermatological, otolaryngological, locomotor, and respiratory diseases. As of 2008, 14,926 subjects aged 45 years or over comprise the Rotterdam Study cohort. Since 2016, the cohort is being expanded by persons aged 40 years and over. The findings of the Rotterdam Study have been presented in over 1500 research articles and reports (see www.erasmus-epidemiology.nl/rotterdamstudy). This article gives the rationale of the study and its design. It also presents a summary of the major findings and an update of the objectives and methods.

## Introduction

The Rotterdam Study was designed in the mid-1980s as a response to the demographic changes that were leading to an increase of the proportion of elderly people in most populations [1]. It was clear that this would produce a strong rise in elderly people living with diseases, as most diseases cluster at the end of life, and that to discover the causes of diseases in the elderly one would have to study risk factors of those diseases [2]. A major approach to finding causes is the prospective follow-up study, which has proven quite effective in finding causes of heart disease and cancer.

## The design of the Rotterdam Study

**Fig. 1:**
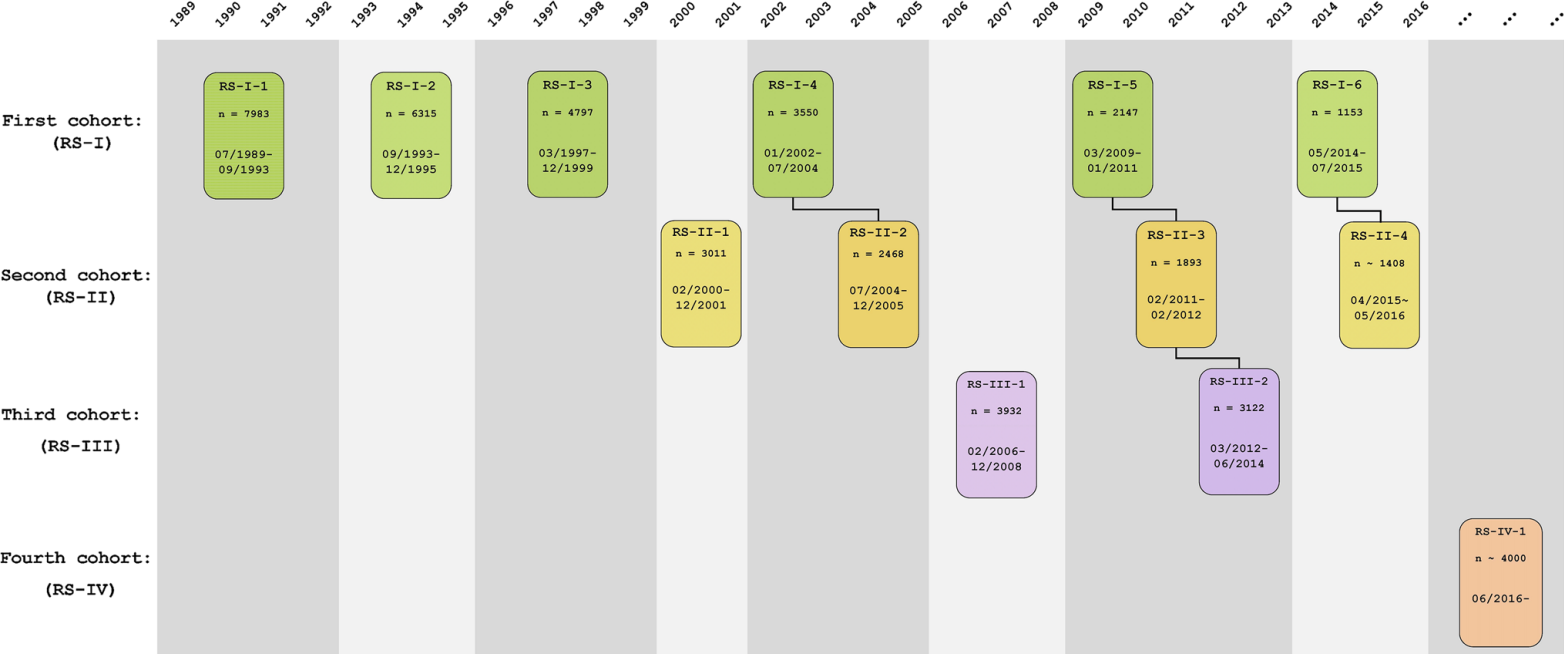
Diagram of examination cycles of the Rotterdam Study (RS). RS-I-1 refers to the baseline examination of the original cohort (pilot phase 07/1989–12/1989; cohort recruitment 01/1990–09/1993). RS-I-2, RS-I-3, RS-I-4, RS-I-5, and RS-I-6 refer to re-examinations of the original cohort members. RS-II-1 refers to the extension of the cohort with persons from the study district that had become 55 years since the start of the study or those of 55 years or over that migrated into the study district. RS-II-2, RS-II-3, and RS-II-4 refer to re-examinations of the extension cohort. RS-III-1 refers to the baseline examination of all persons aged 45 years and over living in the study district that had not been examined already (i.e., mainly comprising those aged 45–60 years). RS-III-2 refers to the first re-examination of this third cohort. Examination RS-I-4 and RS-II-2 were conducted as one project and feature an identical research program. Similarly, examinations RS-I-5, RS-II-3, and RS-III-2 share the same program items. Also, examinations RS-I-6 and RS-II-4 are conducted as one project. RS-IV-1 refers to the baseline visit of a new cohort, established in summer 2016

The design of the Rotterdam Study is that of a prospective cohort study among, initially, 7983 persons living in the well-defined Ommoord district in the city of Rotterdam in The Netherlands (78% of 10,215 invitees). They were all 55 years of age or over and the oldest participant at the start was 106 years [3]. The study started with a pilot phase in the second half of 1989. From January 1990 onwards participants were recruited for the Rotterdam Study. Figure 1 gives a diagram of the various cycles in the study. In 2000, 3011 participants (out of 4472 invitees) who had become 55 years of age or moved into the study district since the start of the study were added to the cohort. In 2006 a further extension of the cohort was initiated in which 3932 subjects were included, aged 45–54 years, out of 6057 invited, living in the Ommoord district. By the end of 2008, the Rotterdam Study therefore comprised 14,926 subjects aged 45 years or over [4, 5]. The overall response figure for all three cycles at baseline was 72.0% (14,926 of 20,744). Since summer of 2016, another extension has started that includes all participants aged 40 years and over. The recruitment of this extension is expected to be completed in 2019 and yield around 4000 new participants.

The participants were all examined in some detail at baseline. They were interviewed at home (2 h) and then had an extensive set of examinations (a total of 5 h) in a specially built research facility in the centre of the district. These examinations focused on possible causes of invalidating diseases in the elderly in a clinically state-of-the-art manner, as far as the circumstances allowed. The emphasis was put on imaging (of heart, blood vessels, eyes, skeleton and later brain) and on collecting biospecimens that enabled further in-depth molecular and genetic analyses. These examinations were repeated every 3–4 years in characteristics that could change over time. There were examination cycles from 1990 to 1993, from 1993 to 1995, from 1997 to 1999, from 2000 to 2001, from 2002 to 2004, from 2004 to 2005, from 2006 to 2008, from 2009 to 2011, from 2011 to 2012, from 2012 to 2014, from 2014 to 2015, and from 2015 onwards (Fig. 1). In spring 2016, the fourth examination cycle for the second cohort (RS-II-4) was finished. In summer 2016 a fourth cohort was established. The age range for this new cohort is predominantly 40–55 years, the anticipated number of participants is 4000.

The participants in the Rotterdam Study are followed for a variety of diseases that are frequent in the elderly: coronary heart disease, heart failure and stroke, Parkinson disease, Alzheimer disease and other dementias, depression and anxiety disorders, macular degeneration and glaucoma, COPD, emphysema, liver diseases, diabetes mellitus, osteoporosis, dermatological diseases and cancer.

The Rotterdam Study has been approved by the institutional review board (Medical Ethics Committee) of the Erasmus Medical Center and by the review board of The Netherlands Ministry of Health, Welfare and Sports. The approval has been renewed every 5 years, as well as with the introduction of major new elements in the study (e.g., MRI investigations).

In the remainder of this article the objectives and major findings will be presented with an update of the research methods for cardiovascular diseases, dermatological diseases, endocrine diseases, liver diseases, neurological diseases, ophthalmic diseases, psychiatric diseases, respiratory diseases, as well as for genetic and biomarker studies and for pharmaco-epidemiologic studies. The emphasis is on major findings from the preceding 2 years (since the previous update paper [6].

For relevant recent EJE references see [6–24].

## Cardiovascular diseases

### Objectives

Research on the epidemiology of cardiovascular disease focuses on the etiology, prediction, and prognosis of cardiovascular disorders (including coronary heart disease, stroke, and heart failure), type 2 diabetes (T2D) and metabolic syndrome. The main emphasis is on prevention and management of a first cardiovascular event but prevention of secondary events is also an area of interest. Putative risk factors include five groups: lifestyle factors, endocrine factors, factors involved in hemostasis, inflammation and endothelial function, metabolomic factors and genetic factors. We have five specific focused themes:

1. *Lifestyle* focused on evaluating the role of lifestyle factors (including nutrition, physical activity, sleep and smoking) in maintaining cardiovascular health as well as the interactions that lifestyle factors might have on other factors (e.g. genes, epigenetic marks and medications).
2. *Biomarkers and genes* aimed to identify relevant biomarkers for the identification of novel mechanisms of disease. These incorporate both molecular and genetic factors together with their potential interactions. Genomics, epigenetic marks and metabolomics play a key role.
3. *Prediction and women’s cardiovascular health* aimed to improve the identification of individuals at increased risk of developing cardiovascular disease in order to point out windows of opportunities that could permit early preventive interventions and personalised care. A special focus is given to evaluating specific factors and formulating targeted strategies to prevent cardiovascular disease in women.
4. *High risk* focused on predictors and prognosis of chronic cardiovascular conditions, like heart failure, pulmonary hypertension, and atrial fibrillation.
5. *Imaging* this work theme aims to identify the contribution that new technologies can provide to the maximum benefit of early diagnosis and accurate prognosis. Major focus is on non-invasive assessment of atherosclerosis to improve the understanding of the atherosclerotic process and the prediction of cardiovascular disease, including measurement of coronary calcification with electron-beam and multi-detector CT (MDCT) and carotid plaque characterization by MRI.

### Major findings

#### Anthropometrics and cardiovascular disease

We evaluated different anthropometric measures, including body mass index, waist circumference, waist to height ratio, waist to hip ratio and a body shape index in association with all-cause, cardiovascular and cancer mortality. We have shown that among different anthropometric measures, a body shape index (ABSI) was strongly associated with the risk of all-cause, cardiovascular and cancer mortality [25]. In contrast to body mass index (BMI) and waist circumference (WC), ABSI showed a differential association with fat mass and fat-free mass in men, but not in women. This could suggest ABSI as a useful tool for identifying men at higher risk of sarcopenic obesity [26]. While the role of BMI for prediction of CVD among the elderly remains controversial, we found that the presence of obesity without metabolic syndrome did not confer a higher CVD risk in the Rotterdam Study. However, metabolic syndrome was strongly associated with CVD risk, and was associated with an increased risk in all BMI categories [27]. We also observed that while obesity had no effect on total life expectancy in older individuals of the Rotterdam Study, it increased the risk of having CVD earlier in life and consequently extended the number of years lived with CVD [28]. Furthermore, among individuals who developed CVD during follow-up in the Rotterdam Study, we identified 3 distinct BMI trajectories. These trajectories marked 3 distinct groups of “stable weight”, “progressive weight gain”, and “progressive weight loss” during follow-up. Other cardiovascular risk factors including glucose and lipid levels differed between the identified BMI subgroups, further highlighting that CVD is a heterogeneous disease with different pathophysiological pathways [27]. Within the European Network for Genetic and Genomic Epidemiology (ENGAGE) consortium, using a mendelian randomization approach, we found that adiposity, as indicated by body mass index, has a causal relationship with coronary heart disease, heart failure and for the first time, ischemic stroke [29]. Also, there were age- and sex-specific causal effects of adiposity on cardiovascular risk factors, including cholesterol, blood pressure, fasting levels of insulin and C-reactive protein [30].

#### Comparison of guidelines

The new American College of Cardiology/American Heart Association (ACC/AHA) guidelines introduced a new cardiovascular (CVD) prediction model and lowered the threshold for treatment with statins to a 7.5% 10-year hard atherosclerotic cardiovascular disease (ASCVD) risk. Using 4854 asymptomatic participants from the population-based Rotterdam Study, we determined the implications of the new ACC/AHA guideline’s treatment threshold and risk prediction model and compared it with the Adult Treatment Panel III (ATP-III), and the European Society of Cardiology (ESC) guidelines. We showed that proportions of individuals eligible for treatment with statins differed substantially among the 3 guidelines [31]. The ACC/AHA guideline would recommend statins for nearly all men and two-thirds of women, proportions exceeding those with the ATP-III or ESC guidelines. All risk prediction models underlying the 3 guidelines provided poor calibration and moderate to good discrimination in our population. To facilitate better clinical decision making, improving risk predictions and setting appropriate population-wide thresholds are necessary.

#### Women’s health

Women experience multiple health issues throughout their life course differently from men. Therefore, attention to women’s health is important in all stages in life. To improve women’s quality of life and guarantee a long-lasting and active role for women in society, prevention of chronic diseases and disability is a key aspect. Our focus, therefore, in the women’s health group is on the major health issues for peri- and post-menopausal women, their risk factors, and prevention strategies [32].

As menopausal health is a crucial aspect in healthy and successful aging, we aimed to characterize a concept for healthy menopause. We conceptualized healthy menopause as a dynamic state, following the permanent loss of ovarian function, which is characterized by self-perceived satisfactory physical, psychological and social functioning, incorporating disease and disability, allowing the attainment of a woman’s desired ability to adapt and capacity to self-manage. Conceptualization of healthy menopause serves as a crucial step in improvement of health in menopausal women, allowing for adapting adequate preventive and treatment strategies [33].

Although cardiovascular disease (CVD) remains one of the leading causes of death and disability for both men and women, our research underscores considerable sex differences in the occurrence of the various manifestations of CVD. Using the long term follow-up from the prospective population based Rotterdam Study, we showed that despite similar lifetime risks of CVD at age 55 for men and women, considerable differences in the first manifestation exist. Men are more likely to develop coronary heart disease as a first event, while women are more likely to have cerebrovascular disease or heart failure as their first event, although these manifestations appear most often at older ages [34]. Since strategies for prevention of stroke and heart failure might differ from strategies for prevention of coronary heart disease, to devise a sex-tailored primary prevention program, knowledge about the first manifestation of CD is important.

#### A gender perspective on health and ageing

Based on 7 domains including chronic diseases, mental health, cognitive function, physical function, pain, social support, and quality of life, we developed a healthy ageing score among women and men in the Rotterdam Study. In all age categories, we found levels of healthy ageing score to be lower in women compared with men. In both genders, the healthy ageing score declined with increasing age, albeit the decline was slightly steeper in women [35]. In an attempt to characterize the relation between fertile life span characteristics and mortality, we found that late first and last reproduction were protective for all-cause mortality, whereas a longer maternal lifespan, postmaternal fertile lifespan, and endogenous estrogen exposure were harmful for all-cause mortality [36]. Further, we used seven metrices of health factors and health behaviors to define the concept of cardiovascular health in the Rotterdam Study. We showed that optimal cardiovascular health was reached by 9.3% of men and 10.4% of women in the Rotterdam Study and was associated with both sex steroids and sex hormone-binding globulin (SHBG) among men and women [37]. To further assess the impact of androgen levels on women’s cardiometabolic health, we formed a multi-center study in which we assessed several cardiometabolic features among women with polycystic ovary syndrome (PCOS), women premature ovarian insufficiency (POI), natural post-menopause women, and women with regular menstrual cycles. This study affirmed the potent effect of androgens on cardiometabolic features, indicating that androgens should indeed be regarded as important denominators of women’s health [38]. Also, we found that women with POI exhibited an unfavorable cardiovascular risk profile, including higher abdominal fat, elevated chronic inflammatory factors, and a trend toward increased hypertension and impaired kidney function compared to premenopausal women of middle age [39].

#### Heart failure and atrial fibrillation

The Rotterdam Study enabled accurate assessment of the incidence and lifetime risk of heart failure and atrial fibrillation in an elderly population [40–42]. It was shown that inflammation and resting heart rate is associated with risk of heart failure [43, 44]. In addition we identified several new risk factors of atrial fibrillation. We found that markers of generalized atherosclerosis in persons without a history of myocardial infarction or angina were associated with a higher risk of atrial fibrillation [45]. Furthermore, high-normal thyroid function [46] and higher levels of dehydroepiandrosterone sulfate, a precursor in the biosynthetic pathway of androgenic and estrogenic sex hormones were associated with incidence of atrial fibrillation [47]. Among individuals free of CVD, we also found an association between epicardial fat, measured by CT scan, with AF that was independent of traditional cardiovascular risk factors, coronary atherosclerosis, left atrial size, and various measures of adiposity [48]. In collaboration with several community-based prospective studies we were able to develop a prediction model for atrial fibrillation, only using variables that are routinely collected in primary care settings [49]. In a large collaborative study as part of the CHARGE consortium, we investigated the genetic variation responsible for 6 traits related to cardiac structure and function. We found two replicated loci for left ventricular dimension and 5 replicated loci for aortic root size [50]. Another topic of interest was the search for genetic determinants of several rhythm and conduction disturbances on the ECG, notably RR-interval, QRS duration, and QT(c)-interval, PR-interval, as well as atrial fibrillation and sudden cardiac death. For example, we identified several new loci for PR interval [51], heart rate [52], and atrial fibrillation [53, 54] in meta-analyses from the CHARGE consortium.

#### Type 2 diabetes

Type 2 diabetes (T2D) has become a global epidemic. We took a comprehensive approach to calculate the lifetime risk of the full range of glucose impairments, from normoglycaemia to prediabetes, type 2 diabetes, and eventual insulin use. At age 45 years, the remaining lifetime risk was 48.7% for prediabetes, 31.3% for diabetes, and 9.1% for insulin use. Our findings highlighted the substantial burden of impaired glucose metabolism on population health, emphasizing the need for more effective prevention strategies [55]. Using multistate life table, we showed that obesity in the middle aged and elderly is associated with a reduction in the number of years lived free of diabetes and an increase in the number of years lived with diabetes [56]. In a mendelian randomization study, we did not find evidence for a causal role of serum gamma-glutamyltransferase on the risk of prediabetes or diabetes [57]. Among inflammatory markers, we found EN-RAGE to be a novel inflammatory marker for pre-diabetes, IL17 for incident T2D and IL13 for pre-diabetes, incident T2D and insulin therapy start [58]. Also we reported that serum apoCIII levels as well as apoCIII-to-apoA1 ratio were associated with incident diabetes independent of known risk factors [59]. ADAMTS13, a novel homeostatic factor, was an independent risk factor for incident prediabetes and type 2 diabetes [60]. In women, we found that low levels of sex hormone-binding globuline and high levels of total estradiol were associated with increased risk of T2D, independent of potential intermediate risk factors such as obesity, glucose and insulin levels [57]. In both men and women, serum dehydroepiandrosterone levels were associated with lower risk of T2D, whereas no associations were found for other hormones in either sex [57, 61]. Further, we provided insights into potential biological mechanisms connecting tobacco smoking to excess risk of T2D by investigating the association between smoking and DNA methylation of genes previously identified for diabetes. We found that tobacco smoking is associated with differential DNA methylation of the diabetes risk genes ANPEP, KCNQ1 and ZMIZ1 [62].

#### Cardiovascular risk factors and prediction

Endocrine, inflammatory and hemostatic factors and risk of coronary heart disease were addressed in several studies. Subclinical hypothyroidism was an independent risk factor of atherosclerosis and myocardial infarction in older women [63]. In a recent study, we compared the change in the accuracy of risk predictions when newer risk markers, representative of various pathophysiologic pathways, were added to the established clinical risk predictors. Among the biomarkers, improvements in coronary heart disease risk prediction were most significant with the addition of amino-terminal pro-B-type natriuretic peptide (NT-proBNP) [64, 65]. Furthermore, plasma C-reactive protein (CRP) and lipoprotein-associated phospholipase A2 (Lp-PLA2) activity were independent predictors of coronary heart disease [66, 67]. Earlier findings included the association of tissue plasminogen activator (TPA) with incident coronary heart disease [68]. Using a comprehensive biomarker assay, we analysed multiple markers of inflammation among 800+ individuals with incident coronary heart disease [69]. We identified EN-RAGE as a novel biomarker for incidence of coronary heart disease, independent of established risk factors and inflammatory markers, such as C-reactive protein [69]. With respect to the prediction of coronary heart disease, EN-RAGE improved prediction significantly indicating that EN-RAGE might be useful in CHD prediction [69]. Regarding novel hemostasis risk factors, we found low ADAMTS13 activity to be associated with increased risk of coronary heart disease, ischemic stroke, and all-cause and cardiovascular mortality beyond the traditional risk factors [70–72]. Recently, we developed and validated a coronary heart disease prediction model tailored for the aging population based on competing risk methodology [73]. Also, we have shown that the non-laboratory based model, based on body shape index, could predict risk of cardiovascular disease as accurately as one that relied on laboratory-based values among men [74].

#### Non-invasive measures of atherosclerosis

Multiple studies focused on the predictive value of non-invasive measures of atherosclerosis for risk of coronary heart disease. Strong associations with risk of coronary heart disease were found for carotid intima-media thickness [75], pulse wave velocity [76], and coronary calcification as assessed by electron-beam CT [77]. The relatively crude measures directly assessing plaques in the carotid artery and abdominal aorta predict coronary heart disease equally well as the more precisely measured carotid intima-media thickness [78]. We also found carotid stiffness to be associated with incident stroke independently of cardiovascular risk factors and aortic stiffness [79]. In persons at intermediate risk of cardiovascular disease, coronary artery calcium provided the best increment in coronary heart disease risk prediction and stratification (to reclassify persons into more appropriate coronary risk categories) [64, 80, 81]. The burden of coronary calcification also provides incremental predictive information for heart failure, but nor for cerebrovascular disease [82, 83]. In a large meta-analysis of 5 population-based studies, including the Rotterdam Study, we showed that coronary artery calcium was present in approximately one-third of women categorized as being at low CVD risk based on the new ACC/AHA guidelines. Presence of coronary artery calcium among low-risk women was associated with an increased risk of CVD and led to modest improvement in prognostic accuracy compared with traditional risk factors [84].

#### Genetic studies

Genetic studies included candidate gene studies [85] and more recently genome-wide association studies of clinical disease and risk factor phenotypes. So far we have contributed to more than 100 Genome-wide association (GWA) studies in the field of cardiovascular disease. These GWA studies are primarily conducted in the framework of the Cohorts for Heart and Aging Research in Genomic Epidemiology (CHARGE) Consortium [86, 87] however in many instances we include further studies. We identified 3 genetic loci associated with uric acid concentration and gout [88]. Three loss-of-function variants in HAL gene were found to associate with histidine levels [89] but not with coronary heart disease. We also identified a significant association between the UMOD gene which encodes Tamm–Horsfall protein and chronic kidney disease [90]. We found four genes for systolic blood pressure, six for diastolic blood pressure and one for hypertension [91–93]. We found multiple loci that influenced erythrocyte phenotypes in the CHARGE Consortium [94]. In a meta-analysis in more than 80,000 individuals from 25 studies, we identified 18 loci for CRP levels. The study highlighted immune response and metabolic regulatory pathways involved in the regulation of chronic inflammation [95]. Novel associations of 12 low-frequency exonic variants with plasma levels of factor VII, factor VIII, and von Willebrand factor were also detected [96, 97]. The association with these variants was independent of the previously identified common variants associated with these traits, and the effect sizes were larger. We performed the first GWA study of ADAMTS13 activity, identifying independent associations with three common variants at the ADAMTS13 locus, as well as one common variant at the SUPT3H locus [98]. Additionally, we used a genotyping array focused on rare exonic variants to identify three independent rare variants in the *ADAMTS13* gene associated with ADAMTS13 activity [98]. We have also identified genetic loci associated with the measures of subclinical atherosclerosis burden. Our genome-wide association studies on the 3 measures of subclinical atherosclerosis identified several new genetic loci [99–101]. Our exome-wide association meta-analysis demonstrated that protein-coding variants in APOB and APOE associate with multiple subclinical atherosclerosis traits as well as clinical coronary heart disease. We have contributed to GWA studies on coronary artery disease [102, 103]. Also, we found that 152 known coronary heart disease SNPs improved the prediction of prevalent but not incident coronary heart disease. This difference may be explained by biases related to the use of prevalent rather than incident coronary heart disease in genome-wide association studies [104]. In addition, by using genome-wide methylation data, we found an effect of tobacco smoking on DNA methylation of 12 coronary artery disease-related genes [105] and associations of blood lipid concentrations with methylation at several metabolic disease-related genes [106], and thus providing novel insights in the pathways underlying cardiometabolic disease.

Thus far, a large number of genetic variants have been identified by GWAS that contribute to the induction and development of cardio-metabolic diseases. Nevertheless, the vast majority of the identified variants map to the non-coding regions of genome that their biological relevant to the disease remain unclear. Non-coding RNAs play regulatory roles in various biological processes and cellular contexts. We identified a number functional variants in microRNA-genes and microRNA binding sites on the 3ÚTR of coding genes that affect miRNA gene regulation and explain some of the observed associations from GWAS of cardio-metabolic phenotypes [107–109].

#### Nutrition and lifestyle

We found that dietary fat intake palmitic acid, which accounts for half of the total saturated fat intake, was associated with an increased risk of coronary heart disease, as was substitution of total saturated fat with animal protein [110]. We did not confirm a consistent association between dietary fat composition and body fat distribution, but we found that total polyunsaturated fatty acids, and in particular n-6 polyunsaturated fatty acids intake, was associated with lower inflammatory profile [111]. We also conducted several studies on the association between nutrition and cancer. We showed that n-3 polyunsaturated fatty acids intake were associated with increased risk of colorectal cancer, but this association was modified by dietary fiber intake [112]. We did find that dietary polyunsaturated fat intake modified the association between total serum cholesterol levels and the risk of colorectal cancer [113, 114]. We also studied whether dietary mineral intake were associated the risk of lung cancer and found that high dietary zinc and iron intake were associated with a reduced risk of lung cancer [115]. In addition to individual nutrient analyses, we performed several studies on a priori and a posteriori *defined dietary patterns* and health outcomes in The Rotterdam Study. For example, we found that adherence to the Dutch dietary guidelines was inversely associated with 20 year mortality in particular due to cardiovascular disease mortality [116]. We also found that a health conscious dietary pattern, characterized by high intake of fruits, vegetables, poultry ranch fish, may have benefits for bone mineral density. Contrary, adherence to a Processed dietary pattern, characterized by high intake of processed meat and alcohol, was associated with lower bone mineral density [117]. Additionally, we evaluated if dietary patterns that explain most variation in bone mineral density and hip bone geometry are associated with fracture risk. We observed that a pattern high in fruit, vegetables and dairy could be associated with lower fracture risk because of high bone mineral density [118].

As part of the CHANCES consortium, we found that adherence to a healthy diet was not associated with cognitive decline [119] but that adherence to the WCRF/AICR Dietary Recommendations for cancer prevention was associated with a lower risk cancer in older individuals, in particular colorectal and prostate cancer [120].

For physical activity, we observed that higher levels of physical activity were associated with increased life expectancy and more years lived without CVD. Of the different types of physical activity included in the study, cycling provided high effects in both men and women [121]. In line with these results, during a 15-year follow-up, it was observed that high physical activity was associated with less coronary heart, mainly explained by cycling and domestic work [122]. Furthermore, it was observed that sedentary behavior was, independent of other physical activity, a risk factor for all-cause mortality [123].

### Methods update

#### Clinical follow-up

Information on clinical cardiovascular outcomes is collected through an automated follow-up system. The follow-up system involves linkage of the study base to digital medical records from general practitioners in the study area and subsequent collection of letters of medical specialists and discharge reports in case of hospitalisation. With respect to the vital status of participants, information is also obtained regularly from the municipal health authorities in Rotterdam. After notification, cause and circumstances of death are established by questionnaire from the treating physicians. Clinical cardiovascular outcomes are adjudicated according to established definitions based on international guidelines by study physicians and medical specialists in the field affiliated with the Rotterdam Study. Methods of follow-up data collection, adjudication of events, and definitions of cardiovascular end points have been described in detail previously in this journal [124]. Systematic follow-up data collection is done for the occurrence of cardiovascular mortality, coronary heart disease (including coronary death, myocardial infarction, and coronary revascularization procedures), heart failure, atrial fibrillation, and sudden cardiac death [124]. Diabetes mellitus is defined based on guidelines of the American Diabetes Association and the World Health Organization. We defined incident diabetes as fasting plasma glucose level ≥ 7.0 mmol/L, or the use of oral antidiabetic medication or insulin, or treatment by diet and registered by a general practitioner as having diabetes.

#### Cardiovascular risk factors

Besides traditional cardiovascular risk factors, five major groups of putative risk factors for cardiovascular conditions are examined. The first group are lifestyle factors, including dietary factors, physical activity, smoking, sleep and vitamin D (as described above). The second are endocrine factors, including diabetes, sex hormones, thyroid gland and adrenal gland hormones and natriuretic peptides (e.g. [46, 47, 63–65]). The third group comprises factors involved in hemostasis, inflammation and endothelial function (e.g. [66, 125, 126]). The fourth group covers genetic factors. In addition to the candidate gene approach, studies are more recently conducted through the genome-wide association approach (e.g. [50–54, 88, 90–95, 99–103, 125, 127–129]). In genome-wide association studies, data from the Rotterdam Study are often combined with those from other studies in the context of the large collaborative CHARGE consortium [86, 87]. Within the fifth group we are applying both proton Nuclear Magnetic Resonance (^1^H NMR) and Mass Spectrometry (MS) for metabolic profiling in 2000 participants of the Rotterdam Study including nearly 200 incident cases of coronary heart disease. Furthermore, in this context, special attention has been given to the contribution of different risk factors in relation to cardiovascular disease in women. Data has been collected to evaluate the impact of specific periods of potential vulnerability across a woman’s lifespan; menarche, pregnancy, and menopause. Also, DNA methylation can regulate gene expression without altering the underlying DNA sequence and is now emerging as a promising molecular strategy for risk stratification for complex disease, including cardiovascular disease. Using the Illumina Infinium HumanMethylation450 array, we have generated DNA methylation profiles of ~ 480,000 CpG sites in In ~ 1000 samples of the RS-III.

#### Non-invasive measures of atherosclerosis

At baseline and follow-up examinations, ultrasonographic assessments of carotid intima-media thickness and carotid plaques were conducted in all participants [75]. At these examinations, also measurements of the ankle-brachial index and aortic calcification (on X-rays of the lumbar spine) were obtained [78]. Carotid–femoral pulse wave velocity, a measure of aortic stiffness, was measured in all *participants of RS-I-3, RS-II-1, and RS-III-1 with an automatic device [76]. Measurements of coronary calcification by electron-beam CT and more recently by MDCT were conducted from 1997 onwards in RS-I and RS-II [77, 80]. From 2003 to 2006, MDCT was used to also quantify calcification in the aortic arch and carotid arteries in RS-I and RS-II. Measurement of carotid plaque components using MRI was done from 2007 to 2012 in all participants from RS-I, RS-II and RS-III with carotid wall thickening on conventional carotid ultrasound. Repeated MRI measures over time were obtained in RS-I and RS-II.

#### Electrocardiographic, echocardiographic and other ultrasound measurements

At every exam, a 12-lead 10-s resting ECG is made and processed by the Modular ECG Analysis System (MEANS) to obtain a series of ECG measurements [130]. Abdominal aortic diameters were measured by ultrasound at RS-I-1, and from 2002 (RS-I-4) onwards in all three Rotterdam Study cohorts. Also from 2002 onwards (RS-I-4), repeated echocardiographic measurements are conducted of structural and functional left heart parameters [131]. From 2009 (RS-I-5) onwards, measurements of structure and function of the right heart are also collected, including estimates of pulmonary artery pressure. In the same round a 3-min resting ECG was measured in all participants.

#### Nutrition and lifestyle

Dietary intake data have been collected in RS-I-1, RS-I-5, RS-I-6RS-II-1, RS-II-3, RS-II-4, and RS-III-1 by using semi-quantitative food-frequency questionnaires (FFQ). In RS-I-1 and RS-II-1, participants completed a checklist about foods and drinks they had consumed at least twice a month during the preceding year and a standardized interview using a validated 170-item semi-quantitative FFQ [132]. For the later waves and cohort, a more comprehensive 389-item FFQ was used during the visits as described in detail previously [133–136]. For all cohorts, nutrient intake data were calculated using the Dutch Food Composition Tables, in close collaboration with the Department of Human Nutrition, Wageningen University, the Netherlands. In RS-I-III, RS-I-5, RS-II-3 and RS-III-I, physical activity data was assessed by means of an adapted version of the Zutphen Physical Activity Questionnaire and the LASA Physical Activity Questionnaire [137–139]. The questionnaire contained questions on walking, cycling, gardening, diverse sports, hobbies and on housekeeping. According to time spent in light, moderate and vigorous activity, metabolic equivalents of task were calculated. Furthermore, we are implementing objective measurement of physical activity with triaxial accelerometers in all participants.

#### Frailty index

As a proxy for overall health we developed a frailty index for the Rotterdam Study, based on predefined criteria [140]. A frailty index is based on the accumulation of health deficits, which can include an unspecified number of symptoms, diseases, laboratory measurements or disabilities, as long as they are health and age related [141]. The severity of frailty is represented by the number of deficits and is expressed on a continuous frailty index score, calculated as the ratio of the deficits present to the total number of variables considered (range 0–1). We calculated a frailty index based on 45 health-related variables, related to cognition, functional status, diseases and biomarkers, for over 11,000 participants. The frailty index showed good construct and criterion validity (e.g. strong association with mortality) [142].

For additional EJE references please see [27, 143–165].

## Dermatological diseases

### Objectives

Dermatoepidemiologic research in the Rotterdam Study focuses on the frequency of the most common skin conditions as well as on genetic and environmental factors associated with these skin diseases. The emphasis is on cutaneous malignancies such as basal and squamous cell carcinomas (BCC and SCC, respectively) and their precursor lesions (actinic keratosis), inflammatory dermatoses such as eczema and psoriasis, and varicose veins. Also, we examine the frequency and determinants including genetics and environmental exposures of skin aging (pigmentation, wrinkling and photodamage) and other visible traits in collaboration with the department of Genetic Identification. Recently, we have introduced optic measures of UV exposed and non-exposed to assess whether they can function as biomarkers of skin and internal diseases.

### Methods

In 2010, dermatology studies were introduced in the Rotterdam Study. To the home interview several items have been added questioning ultraviolet light exposure, history of (personal and familial) psoriasis, history of skin cancer, the diagnostic criteria for atopic eczema, adjusted diagnostic criteria for psoriatic arthritis. More recently, items on skin care and seborrheic dermatitis/dandruff were added.

A full body skin examination by physicians trained in dermatology with a focus on the most common skin diseases is the core contribution of dermatology. The clinical presence and extent of specific skin diseases (i.e., actinic keratosis, malignancies, psoriasis, seborrheic dermatitis, xerosis, hand and flexural eczema, alopecia, and signs of chronic venous insufficiency based on the ‘C’ of the CEAP classification) at time of examination is assessed in a standardized fashion. Other dermatological diseases will just be noted.

The extent of skin aging as a global score and broken down in different aspects such as wrinkling, pigmentary spots, and teleangiecatsia are scored using a validated photonumeric scales and computer algorithms. The Norwood-Hamilton classification and the Ludwig classification is used for male and female pattern hair loss, respectively. Fully standardized 3-dimensional photographs (Premier 3dMDface3-plus UHD, Atlanta, USA) of the face are taken to further assess skin characteristics including sagging, wrinkling at different sites, teleangiectasia and pigmented spots. The colour of the facial skin and at the inner side of the upper arm are measured using a spectrophotometer (Konica Minolta Sensing, spectrophotometer CM-700d, Singapore). Recently, we have included a screening venous ultrasound examination of the lower extermities assessing the deep and superficial venous system. Also, we added skin swabs of the nasolabial fold to investigate the diversity of the microbioom across a large population and assess its relationship with other (skin) diseases.

As for other cancers, pathology data of the cutaneous malignancies is obtained from linkage to the national cancer registry and the Dutch pathology database (PALGA). In a further attempt to identify cohort members with psoriasis, medical files and dispenses at pharmacies have been investigated resulting in over 350 psoriasis cases.

### Major findings

In the first follow-up study including the skin examinations of more than 2000 cohort members, showed that actinic keratosis is very common in this elderly population (AK prevalence was 49% for men and 28% for women) [166]. After adjusting for other factors, baldness in men was associated with a strongly increased risk of actinic keratosis.

A recent update yielded more than 1500 participants with a history of BCC, 450 with a SCC and 150 with a melanoma. We have demonstrated that approximately 30% of people with a BCC develop multiple tumors with 5 years and have developed a prediction model to identify these high risk patients [167]. A first genetic analysis could not confirm any of the existing BCC polymorphisms to be associated with the development of multiple BCC [167]. A subsequent GWAS in an international consortium could not observe the association between common variants and multiple keratinocytic cancers [168]. In new and bigger international collaboration these findings are being re-evaluated. We have presented the first GWAS on actinic keratosis [169]. Several skin color genes such as IRF4, MC1R, ASIP and BCN2 were significantly associated with these premalignant skin lesions independently from skin color. Using compound heterozygosity analysis, several other pigment related genes were identified for AK [170].

In a candidate gene study in almost 6000 people, we confirmed known and identified new variants associated with digital skin colour extraction. Of the two new skin color genes, the genetic variants in UGT1A were significantly associated with hue and variants in BNC2 were significantly associated with saturation [171]. In the International Visible Trait Genetics Consortium, we identified novel pigmentation genes confirmed by functional follow up [172]. Several pigmentation genes were also significantly associated with the presence of pigmented facial spots in a GWAS [169].

Among over 3000 individuals several components of skin aging have been investigated. The most recent finding is a study showing that Individuals carrying the homozygote MC1R risk haplotype looked on average up to 2 years older than non-carriers MC1R [173]. Also, we have demonstrated that digitally extracted wrinkle area from facial 3D photo’s was higher in men (median 4.5%, [interquartile range (IQR): 2.9–6.3]) than in women (3.6%, [IQR 2.2–5.6]). Age was the strongest determinant, and current smoking and lower body mass index were also statistically significantly associated with increased wrinkling. Pale skin color showed a protective effect and, in men, sunburn tendency was associated with less wrinkling. In women, low educational levels and alcohol use associated with more wrinkling, while female pattern hair loss and a higher free androgen index were associated with less wrinkling [174].

The psoriasis patients within the Rotterdam Study have predominantly mild disease. The distribution of subclinical artherosclerosis measures as well as the cardiovascular events were comparable between the 262 psoriasis patients and the reference population [175]. However, psoriasis patients were significantly more likely to have signs of nonalcoholic fatty liver disease based on ultrasonography than their controls after adjusting for potential confounders [176]. Moreover, psoriasis patients were more likely to have liver fibrosis than controls comparing Fibroscan data [177].

## Endocrine diseases

### Objectives

The main objective of the programme of endocrine epidemiology research is to study frequency and etiology of major disorders of the endocrine glands (pituitary, reproductive, thyroid, parathyroid, adrenal, and neuro-endocrine pancreas). These include diabetes mellitus, hypo- and hyper-thyroidism. The evaluation of risk factors for the above mentioned conditions includes serum measurements (such as classical hormones and other endocrine molecules), and genetic determinants of endocrine diseases and traits. In addition, consequences of these endocrine disorders are studied in relation to mortality and aging related diseases, including cardiovascular disease, eye diseases, skin diseases, neurocognitive decline and cancer.

### Major findings

We demonstrated that high-normal thyroid function is associated with an increased risk of atrial fibrillation [46] and subsequently showed that higher FT4 levels are associated with an increased risk of sudden cardiac death, even in euthyroid participants [178]. The absolute 10-year risk of SCD in euthyroid participants increased from 1 to 4% from low-normal to high-normal FT4 levels. A higher thyroid function does not only have negative consequences for the cardiovascular system, since we also showed that a higher thyroid function is associated with increased risk of kidney function decline [179], an increased risk of any solid, lung, and breast cancer [180], as well as an increased risk of AMD [181]. Finally, a high and high-normal thyroid function is also associated with increased risk of developing depression in the elderly [182] and with an increased dementia risk [183]. Interestingly, thyroid function is not related to vascular brain disease as assessed by MRI, suggesting a role for thyroid hormone in nonvascular pathways leading to dementia.

Whereas these data suggest that a higher thyroid function can be detrimental during the aging process, other studies have shown negative consequences of a lower thyroid function as well. We recently showed that a lower thyroid function is associated with an increased risk of NAFLD [184], as well as that a low and low-normal thyroid function are risk factors for incident diabetes, especially in individuals with prediabetes [185]. IN previous studies we already demonstrated that subclinical hypothyroidism is also an independent risk factor of atherosclerosis and myocardial infarction in older women [63]. Also for gait, both low and high thyroid function are associated with alterations in Global gait, Tandem, Base of support and velocity [186].

Future studies will focus on the challenge of defining optimal thyroid function for relevant clinical outcomes and determine which subgroups need specific reference ranges. As part of the Thyroid Studies Collaboration, we recently published four individual-participant data analyses. By analyzing individual participant data from 13 prospective cohorts (70,298 participants) we demonstrated that subclinical hyperthyroidism is associated with an increased risk of hip and other fractures, particularly among those with TSH levels of less than 0.10 mIU/L and those with endogenous subclinical hyperthyroidism [187]. An analysis combining data from 17 cohorts and lead by the Rotterdam Study did not show a higher risk of stroke with subclinical hypothyroidism except in participants younger than 50 years of age [188], whereas higher levels of TSH within the reference range may decrease the risk of stroke [189]. A combined analysis in 14 cohorts focusing on risk of coronary heart disease showed no relationship of TSH levels within the reference range and risk of CHD events or CHD mortality [190].

Much of the work of this research is made possible by large-scale collaboration in consortia, some of which focus on one particular disease or trait while others are more broad spectrum strategic collaborations (e.g., CHARGE, ENGAGE). We are part of several such large consortia studying genetic and epidemiological risk factors for diabetes (MAGIC), and thyroid disease (CHARGE and TSC).

### Major GWAS findings

The main factors that influence the relationship between thyroid hormone and concentrations of TSH in our population-based cohort study are age, smoking, BMI, TPOAb levels, and common genetic variants [191]. In a meta-analysis of GWAS data on TSH levels and free T4 levels derived from up to 26,000 subjects, 26 loci were identified explaining 2–5% of the genetic variation of TSH and fT4 respectively [192]. There was only limited overlap between the loci for TSH and fT4, and evidence was obtained for 5 loci to have sex-specific effects. A GWAS meta-analysis focusing on TPO autoantibodies (an important clinical marker for the detection of early AITD) in 16 cohorts identified five newly associated loci, three of which were also associated with clinical thyroid disease. With these markers we identified a large subgroup in the general population with a substantially increased risk of TPOAbs [193]. A follow-up study identifying 4 additional loci associated provided further insight into the genetic underpinnings of hypothyroidism. A Genetic Risk Score showed strong and graded associations with markers of thyroid function and disease in independent population-based studies [194].

### Methods update

Several specific biomarker assessments in blood/serum/plasma and urine are done for the diagnosis and evaluation of risk factors of endocrine and metabolic diseases (e.g., glucose, TSH, freeT4). Fasting blood samples are collected along with challenged samples as part of a glucose tolerance test. Saliva is collected before and after a dexamethasone-suppression test. Finally, validated questionnaires evaluating nutrient intake (e.g., calcium and vitamins) and activities of daily living, allow to evaluate the role of environmental factors in endocrine conditions and diseases of the elderly.

For additional EJE references please see [195–197].

## Locomotor diseases

### Objectives

The main objective of the program of locomotor epidemiology research is to study frequency and etiology of major disorders of the musculoskeletal system including osteoporosis (OP), osteoarthritis (OA), sarcopenia and chronic musculoskeletal pain. The evaluation of risk factors for the above mentioned conditions includes genomic determinants; serum biomarkers; nutrients; anthropometrics, imaging of bones and joints by X-ray and MRI; and densitometry and body composition quantification by DXA, and pQCT. In addition, these locomotor conditions are studied in the context of other aging related metabolic diseases, including cardiovascular disease and diabetes. Such deep musculoskeletal phenotyping makes the Rotterdam Study a unique resource to study determinants of OP, OA, sarcopenia, and chronic pain and constitutes one of the largest such dataset in the world.

### Major findings

#### Osteoporosis and bone health

We have obtained digitized X-rays for many participants at the several time-points of follow-up, and have applied three different methods to score vertebral fractures: quantitative morphometry (QM), semi-quantitative morphometry (SQ), and the algorithm based qualitative (ABQ) method [198]. A recent comparison of QM assisted by SpineAnalyzer^®^ (SA) software and ABQ, showed that vertebral fracture prevalence differed substantially between the methods, with similar findings being done by the Canadian working group on vertebral fx assessment of the CaMos study. Vertebral deformities misclassified as fractures, typically observed in the SA-QM group classified as mild (Grade 1) inflate drastically the prevalence, and are partly responsible for the observed differences across methods. Re-examining SA-QM grade 1 by assessing endplate depression (the ABQ hallmark) helps discriminating deformities from real fractures. Therefore we proposed this approach to be implemented in radiological clinical practice, thus helping practitioners to assess better the indication of osteoporosis therapy [198].

We determined the relationship of metabolic syndrome and bone health [199] establishing that in contrast to T2D no association with fracture risk was identified despite the fact that, among the metabolic syndrome components, glucose levels were associated with high FN-BMD, highlighting the need to preserve glycemic control to prevent skeletal complications. Further, we have looked at the relationship between uric acid (UA) and bone health outcomes [200] showing how higher levels of serum UA are associated with higher BMD (at the expense of thicker bone cortices and narrower bone diameters) also in interaction with age and vitamin C intake.

Such relationship between bone health and nutritional factors has been extensively examined within the Rotterdam Study. In relation to specific nutrients, we established a plausible favorable relation between high vitamin A intake from the diet with fracture risk in overweight subjects [201]. We also determined that a diet high in acid-forming nutrients (e.g., proteins) may be detrimental to bone health in participants with high intake of dietary fibre [202]. Further, we identified dietary patterns influencing bone health, where beneficial effects on higher BMD were seen with “Health conscious” patterns in contrast to patterns characterized “Processed food” indicate potential susceptibility to presenting low BMD [117]. In addition, we could establish how specific patterns are associated with bone configurations influencing fracture susceptibility [118]. Finally, we developed a food group-based score translated into a BMD-Diet score, capable of profiling groups of food associated with higher/lower BMD levels; of great potential to be adapted in dietary guidelines focused on promoting healthy aging [203].

Although extreme phosphate levels have been associated with mineralization defects and increased fracture risk it was not known whether phosphate levels within normal range are related to bone health in the general population. In the Rotterdam Study we found that serum phosphate was positively related to fracture risk independently from BMD and phosphate intake after adjustments for potential confounders and these findings were replicated in the US Osteoporotic Fractures in Men (MrOS) study [204]. Phosphate and lumbar spine but not femur neck BMD were negatively related in men only. Our findings suggest that higher phosphate levels even within normal range might be deleterious for bone health in the normal population.

#### Osteoarthritis

Over the last years, we have scored X-ray all radiographs of knee, hip and hand of RS I, II and III for osteoarthritic features including up to 20 years of follow-up radiographs. In addition, we have (bilateral) knee MRI images available for a subset (± 1000) individuals of RS III, including a longitudinal follow-up MRI after 6 years. In addition, pain sensitivity measurements have been performed including a quantitative assessment of heat sensitivity on the arm using a standardized device (TSA-II neurosensory analyzer, Medoc), and indications of (wide-spread) pain in any part of the body using a manikin.

Over the last 2 years several established and novel risk factors for OA were examined. No clear association between vitamin serum levels and prevalent, incident or progressive knee, hip or hand OA was observed in the Rotterdam Study and subsequent meta-analysis [205]. We showed for the first time that a marker of tissue inflammation, matrix metalloproteinase-dependent degradation of C-reactive protein (CRPM), predicts the risk of OA progression. This risk was independent of the established biomarkers uCTX-II and COMP [206]. Biomarkers of atherosclerosis were not related to progression of knee osteoarthritis [207]. Furthermore, individuals with cam deformity and those with acetabular dysplasia, two hip shape deformities, were shown to be at higher risk for developing OA; these associations were independent of other well-known risk factors [208]. RNA expression in blood was found to associate with peripheral inflammation in the knee, as measured by joint effusion [209].

A large-scale transcriptome-wide study of muscle strength in human adults identified a total of 221 genes, of which circulating expression levels were associated with muscle strength. This study confirmed associations with known pathways involved in muscle and provides new evidence for over half of the genes identified [210].

#### Chronic musculoskeletal pain

The relationship between the presence of chronic pain and brain volumetrics was studied in the largest study to date. Grey matter volume of the temporal and frontal lobes and the hippocampus were found to be smaller in women with pain compared to those without pain, indicated involvement of emotional processing. The volumetric differences found indicated a sex-specific neuroplasticity in chronic pain [211]. Lower sex hormone levels were found to be associated with chronic musculoskeletal pain, independent from lifestyle and health-related factors in women, suggesting that sex hormones play a role in chronic pain and should be taken into account when a patient presents with chronic pain [212]. Chronic joint pain in the lower body was found to be associated with gait differences independent from radiographic osteoarthritis, indicating that gait assessment may help in identifying individuals with OA from those having pain due to other causes [213]. Indeed, asymptomatic radiographic hip osteoarthritis was found to be associated with gait differences [214] especially in women. Central sensitization, as measured by thermal quantitative sensory testing (QST) was shown be present in community-dwelling elderly individuals suffering from self-reported chronic pain. In addition, several determinants influencing thermal QST measurement were identified [215].

### Major GWAS findings

In a meta-analysis of > 21,000 individuals, we identified six loci to be associated with cartilage thickness, a so-called endophenotype for osteoarthritis [216]. The most prominent four novel associated genetic loci were located in/near TGFA (rs2862851), PIK3R1 (rs10471753), SLBP/FGFR3 (rs2236995), and TREH/DDX6 (rs496547), while the other two (DOT1L and SUPT3H/RUNX2) were previously identified. Exome sequencing data (n = 2050 individuals) indicated that there were no rare exonic variants that could explain the identified associations. This is the first report linking TGFA to human OA, which may serve as a new target for future therapies.

In addition, we identified a variant in the protein-kinase C gene to be associated with neuropathic pain symptoms after total joint replacement highlights [217].

We performed within an international consortium a meta-analysis of GWA studies for whole body lean body mass which consists primarily of skeletal muscle mass, and found five genetic loci to be significantly associated. The loss of lean mass with aging which may lead to a condition called ‘sarcopenia’ is associated with physical disability, falls and fractures, poor quality of life and death [218].

In the field of osteoporosis we identified through leading participation in international consortia less-frequent variants in EN1, the first gene identified combining whole-genome sequencing and GWAS in the field of osteoporosis [219]. Similarly, the Rotterdam Study made part of the first epigenome-wide association study in relation to BMD [220]. Furthermore, we co-lead the discovery of rare coding variants influencing human stature identified in a meta-analysis comprising more than > 700,000 individuals [221].

## Liver diseases

### Objectives

The objective of liver research in the Rotterdam study is concentrated on establishing the prevalence, incidence, risk factors and prognosis of liver diseases in the general population. The two main liver traits of interest are non-alcoholic fatty liver disease (NAFLD) and liver fibrosis. NAFLD is considered the hepatic manifestation of the metabolic syndrome and has become the most common chronic liver disease in Western countries in parallel with epidemics of obesity and type II diabetes mellitus. NAFLD comprises the spectrum from simple steatosis (i.e. fatty liver) to non-alcoholic steatohepatitis (i.e. NASH due to hepatic inflammation), fibrosis, cirrhosis, liver failure and hepatocellular carcinoma. It is estimated that about 25% progress to NASH and more severe stages thereafter [222]. In high-risk populations with metabolic syndrome and obesity, NAFLD appears prevalent in up to 70% [223], a very worrisome trend indeed. Despite over 500 ongoing clinical trials in NAFLD and NASH (www.clinicaltrials.gov), no drug has yet been registered for use in NAFLD patients. Hence the cornerstone of treatment continues to consist of nonspecific life style modifications through weight loss and exercise. We aim to study to what extent the following factors play a role in NAFLD occurring in the general and hence unselected population: components of the metabolic syndrome, obesity, dietary composition, dietary patterns, body composition and sarcopenia, gut microbiome, genetic predisposition and cardiovascular morbidity. With this, we aim to gain more insight into the pathogenesis and provide rationale for more specific life style interventions.

Fibrogenesis of the liver is most probably not only the result of well-known liver diseases, such as viral hepatitis, alcoholic liver disease or NAFLD, but rather a complex interaction between a genetic predisposition and these liver disorders. Liver research in the Rotterdam Study will concern the association between these known causes of liver disease and the occurrence, magnitude, and progression of fibrosis in combination with genetic and environmental factors.

### Methods

#### Abdominal ultrasound

From February 2009 onwards (cohorts RS-I-5, RS-II-3, RS-III-2, RS II-4 and currently ongoing RS-IV-1), trained technicians perform abdominal ultrasonography in Rotterdam Study participants. The liver parenchyma, biliary tract, gall bladder, spleen, pancreas and kidneys are evaluated in combination with Doppler examination of hepatic veins, hepatic artery and portal vein. All images are stored digitally and are reevaluated by an expert hepatologist trained in hepatic ultrasonography.

#### Assessment of steatosis

The diagnosis and grading of liver steatosis is based on ultrasonographic liver brightness, hepatorenal echo contrast, deep attenuation and vessel blurring [224].

Non-alcoholic fatty liver disease is diagnosed by presence of hepatic steatosis on ultrasound and the exclusion of excessive alcohol consumption, presence of viral hepatitis, use of steatogenic agents and recent bariatric surgery.

#### Assessment of fibrosis

Ultrasonographic evaluation of the liver parenchyma and liver surface is performed in order to assess severe fibrosis and/or cirrhosis. Additionally, sonographic signs of portal hypertension are studied (i.e. splenomegaly, venous collaterals, portal vein diameter and flow, hepatic venous flow, and the presence of ascites).

To assess and quantify the grade of fibrosis, trained technicians perform transient elastography in all participants by the Fibroscan^®^. This test measures non-invasively and quantitatively the liver stiffness using an ultrasonic transducer which transmits a vibration wave through the liver. The velocity of the ultrasonic wave is measured in kPa and correlates directly with liver tissue stiffness and ultimately, degree of liver fibrosis [225, 226].

### Determinants of interest

The association between factors known to influence liver function and the occurrence of steatosis and fibrosis are being studied. Additionally, the association of these conditions with age, gender, nutritional intake, concurrent alcohol intake, (risk factors for) viral hepatitis, BMI, waist-to-hip ratio, serum glucose, insulin, and diabetes mellitus, hypertension, serum cholesterol, triglycerides, dietary composition, macronutrients, dietary patterns, sarcopenia, body composition, and gut microbiome are investigated. All clinical information is obtained by interview (updated with liver specific questions) and clinical examination. More recent efforts are focused on identifying common genetic variants associated with liver steatosis and/or fibrosis.

### Main findings

We found a high prevalence of NAFLD of 35.1% within the Rotterdam Study population [227]. Main risk factors for NAFLD were found to be age, decreased physical activity lever, smoking, increased waist circumference, glucose intolerance, hypertension, and hyperlipidemia. Inversely, the risk of NAFLD seems to decrease after statin therapy [228]. Furthermore, using our ultrasound data as reference, we examined the performance of the well-known fatty liver disease index (FLI, based on waist circumference, BMI, triglyceride and gamma-glutamyltransferase (GGT) levels) in the Rotterdam Study population, and found that the FLI is a highly valid tool to predict NAFLD [229]. In another study, we found that all serum liver enzymes are related to all-cause mortality, as well as specifically cardiovascular (GGT) and cancer-related (alkaline phosphatase and aspartate aminotransferase) mortality [230]. Moreover, we have examined the role of genetic factors in the multifactorial etiology of liver fibrosis, and found for example that the single nucleotide polymorphism (SNP) of the interferon gamma receptor 2, a pro-inflammatory gene known to be associated with progression to liver fibrosis in chronic hepatitis C patients, also was related to liver stiffness in the Rotterdam Study participants [231]. Recently, we found that coffee consumption of three cups or more per day, which was found to be beneficial in certain chronic liver diseases and liver fibrosis [232], appeared associated with lower liver stiffness values in the general population as well [233]. At this moment, we are investigating differences in dietary composition (macronutrients) and dietary patterns, body composition and differences in gut microbiota between NAFLD and non-NAFLD participants. Moreover, more studies are currently underway to look at known and unknown genetic and epigenetic factors for liver stiffness and NAFLD.

For additional EJE references please see [234, 235].

## Neurological diseases

### Objectives

Neuroepidemiologic research in the Rotterdam Study focuses on the frequency, etiology and early recognition of the most frequent neurologic diseases in the elderly. We study neurodegenerative diseases (dementia, including Alzheimer disease, and Parkinson disease), cerebrovascular disease (both ischemic stroke and intracerebral hemorrhage as well as transient ischemic attacks), migraine and polyneuropathy. In all of these disorders clinical symptoms typically become manifest late in the disease course, the occurrence of clinical disease does not reflect the underlying spectrum of disease-related pathology, and most of the clinical syndromes are etiologically heterogeneous. Therefore, an additional research focus is on the causes and consequences of pre-symptomatic (brain) pathology that can be assessed with non-invasive modalities, which include MR-imaging, cognitive testing, gait assessment, and electromyography (EMG).

### Major findings

In recent years, we have published contemporary data on incidence of these major neurological diseases. We were the first to show declining incidence of dementia [236] and in recent papers we have demonstrated similar trends for stroke [237] and Parkinson disease [238]. We have also published on prevalence of polyneuropathy [239], showing that 5.5% of the general population suffers from this disease with the disease going unrecognized in almost half of these persons. We have also published normative data for various pre-clinical markers, including cognition [240], gait [241], and various MRI-markers [242–244].

One of the main areas of focus in recent years has been understanding how brain pathology affects motor function, with a special emphasis on gait. We have shown strong and specific association of gait with cognition [245], DTI markers [246] and daily functioning [247]. Ongoing work regarding gait includes its longitudinal associations with clinical diseases, including stroke, dementia and Parkinson’s disease. Interestingly, using a different test we have already shown motor function to be a predictor of dementia onset over a 9 year period [248]. Moreover, we have also made several contributions towards understanding the etiology of Parkinson’s disease [249–251].

Main findings in recent years with respect to stroke and Alzheimer’s disease, include the study of the following determinants: cerebral perfusion [252], thyroid function [189], aortic valve calcification [253], white matter microstructure [254], orthostatic hypotension [255], mid-life blood pressure [256], depression [257], and parental family history [258].

Similarly, we have now published on several determinants of polyneuropathy [259, 260] and migraine [261]. In coming years we will be seeking to develop a research line on epilepsy.

Given our longstanding interest in unraveling the etiology of neurodegenerative diseases, our current work also involves leveraging the longitudinal and repeated data collection from the Rotterdam Study to investigate trajectories of various pre-clinical markers and disentangle the patterns of how those relate to incident disease [262–264].

In the field of neurogenetics, we have contributed to or led several conventional GWAS efforts as well as more state-of-the-art genomics to discover novel genetic loci for neurologic diseases and their endophenotypes [265–269].

Finally, we are actively investigating how findings on etiology of neurologic diseases can be translated towards public health issues on prevention [270, 271] as well as clinical needs regarding prediction [254, 272, 273] and possibly even interventional studies [274].

### Methods update

#### Assessment of dementia and Alzheimer disease

In the baseline and follow-up examinations participants undergo an initial screen for dementia with the Mini Mental State Examination (MMSE) and the Geriatric Mental Schedule (GMS), followed by an examination and informant interview with the Cambridge Examination for Mental Disorders of the Elderly (CAMDEX) in screenpositives (MMSE < 26 or GMS > 0), and subsequent neurological, neuropsychological and neuroimaging examinations [275, 276]. Of subjects who cannot be reexamined in person, information is obtained from the GPs and the regional institute for outpatient mental health care. A consensus panel makes the final diagnoses in accordance with standard criteria (DSM-III-R criteria; NINCDS-ADRDA; NINDS-AIREN).

#### Assessment of Parkinsonism and Parkinson disease

Participants are screened in the baseline and follow-up examinations for cardinal signs of parkinsonism (resting tremor, rigidity, bradykinesia, or impaired postural reflexes). Persons with at least one sign present are examined with the Unified Parkinson’s Disease Rating Scale and a further neurologic exam. PD is diagnosed if two or more cardinal signs are present in a subject not taking antiparkinsonian drugs, or if at least one sign has improved through medication, and when all causes of secondary parkinsonism (dementia, use of neuroleptics, cerebrovascular disease, multiple system atrophy, or progressive supranuclear palsy) can be excluded [277].

#### Assessment of stroke and stroke subtypes

History of stroke at baseline was assessed through interview and verified in medical records. Putative incident strokes get identified through the linkage of the study database with files from general practitioners, the municipality, and nursing home physicians’ files, after which additional information (including brain imaging) is collected from hospital records. A panel discusses all potential strokes and subclassifies strokes into ischemic, hemorrhagic or unspecified [278, 279]. We also systematically collect transient ischemic and neurological attacks [280].

#### Assessment of cognitive function

Global cognitive function is measured through the Mini Mental State Examination (MMSE) in all surveys. From the third survey (RS-I-3) onwards we added a 30 min test battery that was designed to assess executive function and memory function, and which includes a Stroop test, a Letter Digit Substitution Task, a Word Fluency Test, and a 15 words Word List Learning test. This test battery was expanded from the fourth survey onwards (RS-I-4) to include motor function assessment using the Purdue Pegboard Test. Moreover, from 2009 onwards we expanded further by including the Design Orientation Test (DOT) and a modified version of the International Cooperative Ataxia Rating Scale (ICARS), which assess visuo-spatial orientation and ataxia respectively [240, 281, 282].

#### Assessment of gait patterns

Halfway through RS-III-1, we successfully implemented the assessment of gait in all participants using the GAITRite walkway (http://www.gaitrite.com/). Gait is assessed using a 5.79 m long walkway (GAITRite Platinum; CIR systems, Sparta, NJ, USA: 4.88 m active area; 120 Hertz sampling rate) with pressure sensors. Participants perform a standardized gait protocol consisting of three different walking conditions: normal walk, turning and tandem walk. In the normal walk, participants walk over the walkway at their own pace. This walk is repeated four times in both directions (yielding a total of 8 recordings). In turning, participants walk over the walkway at their own pace, turn halfway and return to the starting position (1 recording). In the tandem walk, participants walk tandem (heel-to-toe) over a line visible on the walkway (1 recording). A total of 30 spatiotemporal gait variables are calculated by the walkway software and downloaded offline for further analysis. Subsequently, principal components analysis on these thirty gait variables is performed to derive summarizing factors, referred to as gait domains. The following gait domains are used: Rhythm, Pace, Phases, Base of Support, Variability, Tandem, and Turn. Gait domains can be compared to cognitive domains, in which each domain reflects a different aspect of the overall concept [241]. Since 2 years we have added another walk to our protocol, namely a dual-task walk, in which participants answer a difficult calculation, while walking over the walkway. The aim of this walk is to compare it with the original normal walk, thereby obtaining the amount of central interference and input on gait.

#### Assessment of polyneuropathy

Starting in January 2013, we have successfully implemented a protocol to assess polyneuropathy [239]. This includes a full work-up including questionnaire, neurological exam, and EMG in all participants. In coming years, we will publish on the prevalence, risk factors, and clinical correlates of polyneuropathy in the general population. The continuous measures of conductivity obtained through EMG can also serve as excellent endophenotype for genetic and biomarker studies.

#### Assessment of migraine

Migraine is assessed using a validated questionnaire and includes information of aura, severity, and duration of migraine [283].

#### Rotterdam Scan Study: brain imaging within the Rotterdam Study

In 1991, a random sample of 111 participants underwent axial T2-weighted magnetic resonance (MR) imaging to assess presence and severity of white matter lesions [284]. In 1995, a random sample of 563 non-demented participants underwent brain MR imaging in the context of the Rotterdam Scan Study. From August 2005 onwards (RS-II-2 and further), a dedicated 1.5 Tesla scanner is operational in the research center of the Rotterdam Study, and brain imaging is performed in all study participants without contra-indications [285].

Currently, the follow-up of this latter sample extends to up to 12 years (see further section on population imaging).

For additional EJE references please see [273, 286–303].

## Ophthalmic diseases

### Objectives

Ophthalmic research in the Rotterdam Study focusses on occurrence, causally related determinants, and predictors of common eye diseases. Our main focus is on age-related macular degeneration (AMD), glaucoma, and myopia, and particularly in the last few years we investigated genetic risk variants and pathways. To this end, we connected with many other epidemiologic studies in all parts of the world and formed large international consortia.

### Major findings

#### Age-related macular degeneration (AMD)

AMD has been genetically dissected for the most part, and the past 2 years were geared towards understanding the genetic effects and their role in AMD pathogenesis. With the IAMDGC consortium, we analyzed 33,000 participants and identified 52 independently associated common and rare variants distributed across 34 loci [304]. Many of these loci harbored novel genes, and aside from many common variants, various rare variants were identified. The genes in the complement cascade as well as ARMS2 remained the major genes. A subsequent exercise of IAMDGC was to evaluate pleiotropy of the AMD risk variants, and it was found that at least 16 disorders show substantial genetic overlap with AMD [305]. In our own Rotterdam cohort, we used the findings from IAMDGC to investigate genetic variants in miRNAs and miRNA-binding sites [306]. We identified variants in miRNAs (miR-4513; miR-3591; miR-3135b), and 54 variants in miRNA-binding sites associated with AMD. Experimentally, we showed that miR-210-5p influences expression of CFB. These findings are exciting as they point to potential targets that can control the complement pathway, and halt AMD progression. Apart from genetics, we also studied phenotypic association and course of disease. Together with two other population-based studies (3CC), we found that 19–28% of unilateral any AMD became bilateral in 5 years, and 27–68% of unilateral late AMD became bilateral during that time [307]. Smoking and carriership of genetic risk variants increased progression rates substantially. We also investigated retinal pseudodrusen in more detail, a distinct AMD lesion [308]. 5% of the Rotterdam Study had these lesions, women twice as often as men, as did carriers of certain genotypes.

#### Myopia (nearsightedness)

We prolonged our research in the field of refractive errors and myopia in the CREAM consortium.

This time we performed a joint meta-analysis to test gene-environment interaction effects, and identified six novel loci (FAM150B-ACP1, LINC00340, FBN1, DIS3L-MAP2K1, ARID2-SNAT1 and SLC14A2) associated with refractive error [309]. In Asian populations, three genome-wide significant loci AREG, GABRR1 and PDE10A also exhibited strong interactions with education. These findings clearly show that genes for refractive errors need environmental triggers in order to have a significant effect. We were also interested in the susceptibility period for refractive error genes. We therefore investigated the association between age-of-onset of variants at our previously identified loci and refractive error in various cohorts of different ages, including the Rotterdam Study [310]. Specific variants could be categorized as showing evidence of: (a) early-onset effects remaining stable through childhood, (b) early-onset effects that progressed further with increasing age, or (c) onset later in childhood. This shows that most genes in a complex trait such as refractive error do not have a continuous effect, but rather act during a specific age period. Next steps in myopia research will include gene finding in very large data sets (> 100,000), identification of pathways, and search for leads for intervention.

#### Primary open-angle glaucoma (POAG)

The glaucoma research entailed gene finding as well as the study of the associations with glaucoma parameters. The latter included the study of intraocular pressure (IOP) across Europe in the E3 (N = 43,500) consortium [311]. Higher IOP was observed in men, with higher body mass index, shorter height, higher systolic blood pressure, and more myopic refraction. An inverted U-shaped trend was observed between age and IOP, with IOP increasing up to the age of 60 and decreasing in participants older than 70 years. Gene finding was performed in the IGGC consortium. We conducted a genome-wide association meta-analysis of IOP and optic disc parameters and validated our findings in multiple sets of POAG cases and controls. We identified 9 new loci for vertical cup-disc ratio (VCDR), 1 for IOP, 5 for optic nerve cup area, and 6 for disc area. Some genomic regions affected both IOP and the disc parameters. Furthermore, we identified a novel association between CDKN1A and POAG, statistically as well as functionally in a zebrafish model. We also evaluated sequence variations in the myocilin (MYOC) gene, a gene that accounts for approximately 2–4% of glaucoma cases [312]. Mutation Gln368Stop in this gene is known to increase intraocular pressure. We found that this variant was also very frequent among unaffecteds from The TwinsUK and Rotterdam Study (12.5 and 19.4%, respectively). This showed that this seemingly functional variant may not have such large effects as previously thought. Finally, we investigated the performance of a new reference panel, the Haplotype Reference Consortium (HRC), for imputation of genetic variants [313]. We showed that imputation using the HRC panel improved the concordance between assayed and imputed genotypes at common, and especially, low-frequency variants. HRC imputation significantly improved *P* values for genetic associations with glaucoma parameters, thus our next step is to continue gene discovery using HRC in very large data sets of multi-ethnic origin.

#### Retinal vasculature

We also continued this line of research and investigated the meaning of vessel diameter in the retina for pathology at other parts of the body, in particular the brain [314–318]. Retinal vessel calibers were associated with enlarged perivascular spaces in the brain and with white matter microstructure. Interestingly, it was also associated with survival, vitamin D, and N-Terminal Pro-B-Type Natriuretic Peptide, a protein associated with ischemia. This indicates that retinal vessel diameters are important biomarkers for the vascular status elsewhere in the body, and may predict life expectancy.

### Methods update

At baseline and follow-up examinations, participants undergo ophthalmic measurements including best-corrected ETDRS visual acuity, refractive error, Goldmann applanation tonometry, keratometry, slit lamp examination of the anterior segment, and visual field testing. After pharmacological mydriasis, we make 35° color photographs of the macular area, and 20° simultaneous stereoscopic imaging of the optic disc and macular area using stereoscopic digital imaging (Topcon camera). We image retinal layers at the macula and optic disc with Fourier3D Spectral domain optical coherence tomography (Topcon), measure axial length, and biometry of the cornea, anterior chamber, lens, posterior chamber, and retina with Lenstar (Haag-Streit); and perform fundus autofluorescence, infra-red and red-free measurements with Heidelberg. For the newest cohort (RS4-1), we have added corneal topography measurements (Pentacam; Oculus), and replaced visual field screening by Frequency Doubling Technology C20-2 (Carl Zeiss Meditec). The classification of AMD, POAG, refractive error, and retinal vessel diameters remain unchanged.

For additional EJE references see [311, 319–322].

## Psychiatric epidemiology

### Objectives

The aim of the psychiatric research in the Rotterdam Study is to investigate the determinants, correlates and consequences of common psychiatric problems in the elderly. The focus lies on studies of depressive and anxiety disorders, sleep disturbances, and complicated grief.

### Study design update

Since 1994 (RS-I-2) most participants in the Rotterdam Study are screened for depressive symptoms and from the third examination (RS-I-3), 1997–1999, onwards, depressive disorders have been ascertained systematically. Assessments of anxiety disorders, sleeping disturbances, and complicated grief were added in the subsequent examination (RS-I-4) and have been performed in all follow-up visits of the original and added cohorts. Other additions to the protocol included a screening for psychotic symptoms in one cohort (RS-III) and, from January 2012 to October 2014, ambulatory polysomnography. In a subsample, taedium vitae was assessed. The most recently introduced assessments include sexual activity, aggression and neuroticism.

### Major determinants

Psychiatric research in the Rotterdam Study focuses on biological risk factors. The vascular depression hypothesis was tested with different measures of atherosclerosis, arterial stiffness and cerebral blood flow [323]. We examined whether blood levels of vitamins and fatty acids, immune parameters, and markers of folate metabolism increased the likelihood of depression [324]. Diurnal patterns of cortisol secretion were studied and recently we performed a low-dose dexamethasone test to assess the negative feedback of the hypothalamic–pituitary–adrenal (HPA) axis functioning [325]. Moreover, several GWAs were conducted in collaborative efforts focussing on depressive symptoms, sleep, anxiety and cortisol [326–328]. Several, mostly cross-sectional studies of brain morphology as possible determinants and correlates of common psychiatric disorders were completed [329]. Also, psychiatric problems and psychological traits such as happiness, sleep duration, and depression are increasingly investigated as determinants of health and mortality [330, 331].

### Major clinical outcomes

Information on depression is obtained from (a) psychiatric examinations, (b) self-reported histories of depression, (c) medical records, and (d) registration of antidepressant use [332]. The psychiatric examination during each visit consists of an assessment and screening with the Center for Epidemiologic Studies Depression Scale (CES-D), and in the screen-positive participants a semi-structured interview performed by a trained clinician (Schedules for Clinical Assessment in Neuropsychiatry). To continuously monitor incidence of depression throughout follow-up, trained research-assistants scrutinize the medical records of general practitioners and copy all information mentioning depressive symptoms.

The following anxiety disorders are assessed with a slightly adapted Munich version of the Composite International Diagnostic Interview: generalized anxiety disorder, specific and social phobia, agoraphobia without panic disorder, and panic disorder [333]. In addition, the HADS-A is used to assess anxiety traits continuously.

Sleep quality and disturbance is measured with the Pittsburgh Sleep Quality Index. In addition, sleep duration and fragmentation are assessed with actigraphy, a method that infers wakefulness and sleep from the presence or absence of limb movement [334]. In total, nearly 2000 persons participated in this actigraphy study: they wore an actigraph and kept a sleep diary for, on average, six consecutive nights. Follow-up assessments of actigraphic assessments in these participants have been conducted. Ambulatory polysomnographic (PSG, i.e., full sleep EEG) recordings of one night have been conducted in 940 participants. We scheduled home visits of a research assistant who placed the sensors to record an ambulant PSG (Vitaport 4; Temec, Kerkrade, the Netherlands). The PSG included six EEG channels, bilateral electrooculography, electromyography, electrocardiography, respiratory belts on the chest and abdomen, oximetry, and a nasal pressure transducer and oronasal thermocouple to measure airflow [335]. All recordings were scored according to American Association of Sleep Medicine guidelines by a registered Sleep Technologist. Recordings were manually scored in 30- s epochs for identification of sleep stages; each epoch was scored as Wake, N1, N2, N3 or REM sleep. In addition, we used PRANA (PhiTools, Strasbourg, France) software to automatically measure the microstructure of sleep, e.g. spindles and REM density. Polysomnography recordings are also used to calculate the apnea–hypopnea index.

Circadian rhythms: Sleep–wake activity patterns over a week are studied with actigraphy As a marker of circadian rhythms. In more than 1700 persons we calculated interdaily stability, i.e. the stability of the rhythm over days and the intra-daily variability, i.e. the fragmentation of the rhythm [336].

The Inventory of Complicated Grief is used to identify traumatic grief. This is a condition distinct from normal grief and bereavement-related depression, characterized by symptoms like disbelief about the death and searching for the deceased.

### Major findings

*Depression* In a series of studies we found some evidence for the vascular depression hypothesis. More severe coronary and extra-coronary atherosclerosis were associated with a higher prevalence of depression, as were cerebral haemodynamic changes [323]. However, our data did not support a specific symptom profile of vascular depression as previously defined. Most importantly, we found no longitudinal relation between peripheral atherosclerosis and incident depression [337]. Recently, we prospectively studied cerebral vascular risk factors such as white matter lesions, silent infarcts or blood flow in relation to depression [338]. We found evidence that small vessel disease predicted the onset of depression. This suggests that atherosclerotic processes in the brain are a specific risk factor for depression.

*Sleep* We investigated the relationships of sleep duration with both cardiovascular risk factors and psychiatric disorders. We also aimed to explain sex differences in subjective and actigraphic sleep parameters [339]. If assessed by diary or interview, elderly women consistently reported shorter and poorer sleep than elderly men. In contrast, actigraphic sleep measures showed shorter and poorer sleep in men. These discrepancies were partly explained by sleep medication use and alcohol consumption. The first results using polysomnography to measure sleep EEG suggest that REM-density is a marker of depressive symptoms in the general population [335]. Other results suggest that sleep apnea and depressive symptoms are not related, although both result in fatigue [340].

*Anxiety* We studied anxiety as a determinant of mortality and cardiovascular disease, and found anxiety in the elderly does not predict physical morbidity independent of baseline health and behaviour. In contrast [341], we could show that mild cognitive impairment is associated with incident anxiety disorders [342].

*Complicated grief* In our population-based study of 5741 elderly persons, current grief was reported by 1089 participants, of these 277 (25 or 4.8% of total) were diagnosed with complicated grief, the vast majority of which had no clinical symptoms of anxiety or depression. Persons with complicated grief were older, had a lower level of education, and more often had lost a child [343]. Recently published work suggests that complicated grief occurs together with structural brain atrophy more often than expected by chance [344].

*Sexual activity* Almost half of partnered older adults engage in sexual activity and over two-thirds engage in physical tenderness, but very few unpartnered older adults engage in sexual behaviour [345]. The greatest barrier to being sexually active at older age is lack of sexual partner availability, for which women are particularly disadvantaged. Moreover, sexual activity is strongly determined by well-being, in particular happiness rather than lack of depression [346].

*Genetics of common psychiatric disorders* In the past years, we have performed a series of genome-wide association studies of the above psychiatric and psychological phenotypes, mostly as part of the CHARGE consortium and more recently as part of the Psychiatric Genetics Consortium. While initial analyses yielded no convincing genome wide significant results as studies were strongly underpowered, more recent work with larger sample sizes led by our group in the CHARGE or as part of the PGC consortium shows promising results for depression and depressive symptoms [326].

Finally, ongoing psychiatric research projects examine whether and how psychological well-being or psychiatric problems contribute to survival. Most importantly, we are interested in whether the effects are specific to certain behaviour or emotions, are independent of confounding by physical disease, or can be explained by lifestyle, immunological or hormonal regulation [347].

For additional EJE references see [348–353].

## Respiratory diseases

In the Rotterdam Study (RS) we investigate the prevalence and incidence of respiratory diseases in middle-aged and older adults, and aim to elucidate the genetic, environmental and life style risk factors for the occurrence of these diseases. Moreover, by applying systems genetic and systems biology approaches, we aim to decipher the pathogenesis and pathophysiology of respiratory diseases. The main focus of research of the respiratory epidemiology group is on common obstructive airway diseases, encompassing asthma, ACOS (Asthma COPD Overlap Syndrome) and Chronic Obstructive Pulmonary Disease (COPD), but also respiratory infections, pneumonia, pulmonary hypertension and lung cancer are thoroughly investigated. Lung function measurements encompassing spirometry and diffusion capacity are performed in all participants during the research centre visit of the RS using a Master Screen^®^ PFT Pro by trained paramedical personnel according to ERS/ATS Guidelines [5, 354].

### Lung function and Chronic Obstructive Pulmonary Disease (COPD)

In the large prospective population-based RS cohort, we have determined the prevalence and incidence of COPD in older adults according to age, sex and smoking history [355, 356]. In international collaboration, we have elucidated the genetic determinants of the lung function measurements Forced Expiratory Volume in one second (FEV1), Forced Vital Capacity (FVC) and the FEV1/FVC ratio, the defining characteristic of an obstructive syndrome [357–360]. In the most recent genome-wide association study of COPD, we have discovered 22 loci of genetic susceptibility, including 9 loci which have been previously associated with lung function in the general population, and 4 new loci (EEFSEC, DSP, MTCL1 and SFTPD) [359]. Intriguingly, we highlighted that 2 loci associated with COPD (FAM13A and DSP) were shared with pulmonary fibrosis, but had opposite risk alleles. Moreover, using a systems genetics analysis approach, we have discovered the molecular mechanisms underlying variations in lung function [361].

### COPD, co-morbidities and frailty

COPD does not only affect the lungs, but is frequently associated with extrapulmonary manifestations and systemic consequences. Therefore, we have investigated multiple co-morbidities of COPD, encompassing cardiovascular diseases, cerebrovascular diseases (Carotid artery atherosclerotic plaques, cerebral microbleeds and stroke) and osteoporosis [362–367]. Importantly, we have meticulously validated acute exacerbations of COPD in participants with COPD in the RS, and examined the impact of these exacerbations on acute cardiovascular events (e.g. atrial fibrillation, sudden cardiac death), acute cerebrovascular events (stroke), and mortality [367, 368]. Moreover, we have highlighted differences in the distribution of cause-specific mortality in patients with COPD according to disease stage [363, 366].

Frailty is a common geriatric syndrome, characterized by a lack of functional reserve to stressors, and defined by Fried et al. as meeting three or more of five established criteria for frailty (nutritional status, physical activity, mobility, grip strength and exhaustion). Of 2833 RS participants with sufficiently evaluated frailty criteria, 163 (5.8%) participants were frail, whereas the prevalence of frailty was significantly higher in subjects with COPD (10.2%) [369]. Adjusted for age, sex and co-morbidities, frail elderly had a significantly increased risk of dying within 3 years, compared to the non-frail elderly [370]. In subjects with COPD, the prevalence of frailty was highest when they suffered from severe airflow limitation, dyspnea and/or frequent exacerbations. Importantly, COPD elderly who were frail had significantly worse survival [369]. Therefore, COPD is a key component of the chronic disease domain of the Healthy Aging Score, which has recently been developed by the RS investigators [35].

Additional EJE references see [356, 371–374].

## Genomics, biomarker and microbiome studies

### Objectives

The team in this research line focusses on bio-banking activities of the participants of the Rotterdam Study and investigates molecular biological determinants of disease in these specimen (i.e., DNA, RNA, proteins, metabolites, microbes, etc.). Bio-banking involves collecting, storing and managing the biological tissues of participants of the Rotterdam Study at all follow-up measurements. This concerns mainly blood, urine, saliva, hair and faeces but with microbiome studies several other specimens are being collected (such as skin swaps, nose swaps, eye swaps, etc.). We have further stored PBMC’s for the isolation of induced pluripotent stem (iPS) cells. The research focus of this group concerns assessment of biological determinants of disease (biomarkers) in these biomaterials and the analysis of markers using genomic technologies (such as SNP arrays and next generation sequencing (NGS)). The materials and data generated by this research line now sum up to ~ 3 × 10^12^ data-points, and are actively used by all research groups of the Rotterdam Study. An overview of all the “omics” datasets in the Rotterdam Study cohorts is given in Table 1.

**Table 1:** Overview of sample numbers with “omics” datasets across the 3 Rotterdam Study (RS) cohorts with the number and type of measurement for each omic method

Genomics data type	Total	Datapoints/sample		RS I^a^	RS II^a^	RS III^a^
		Number	Type			
GWAS SNP data	11,502	40,000,000	SNPs	6291	2157	3054
Exome array	3183	250,000	SNPs	3183	–	–
Whole exome sequencing (WES)	3778	693,000	Variants	3778	–	–
Whole genome sequencing (WGS)	96	3,000,000	Variants	96	–	–
Genome wide expression (array)	881	25,000	Genes	–	–	881
Genome wide expression (RNA Seq)	829	18,000,000	Reads	–	500	329
Genome wide DNA methylation	1600	450,000	CpG’s	100	500	1000
Telomere length (PCR)	1800	1	–	1800	–	–
Mitochondrial DNA (PCR)	500	1	–	500	–	–
Microbiome 16S rRNA (faeces)	2000	500	OTU’s	–	–	2000
Metabolomics (NMR/UPLC MS)	1826	4000	Metabolites	1826	–	–
Metabolomics (NMR “Nightingale”)	5381	228	Metabolites	2880	663	1838
Serum protein profile^b^	9820	35	Proteins	3812	2542	3466
Total ‘omic’ datapoints in RS:	43,196 × 62,422,765 = 2,696,413,756,940					

*SNP* single nucleotide polymorphism, *CpG* a two-nucleotide position (C next to G on the same strand) of which the C can be methylated; *OTU* operational taxonomic unit

^a^RS1, First cohort of the Rotterdam Study; RS2, Second cohort of the Rotterdam Study; RS3, Third cohort of the Rotterdam Study

^b^Total estradiol, total testosterone, sex hormone-binding globulin, dehydroepiandrosterone, dehydroepiandrosterone sulfate, androstenedione, 17-hydroxyprogesterone, cortisol, corticosterone, 11-desoxycortisol, vitamin D, thyroid stimulating hormone, free T4, interleukins, C-reactive protein, Insulin-like growth factor 1, insulin, iron, ferritin, transferrin, fibrinogen, homocysteine, folic acid, riboflavine, pyridoxine, SAM/SAH ratio, cobalamine, Lp-PLA2, Fas/Fas-L, abeta42/40

### Major findings

Rotterdam Study investigators are playing leading roles in several of the large global consortia focused on assessing the contribution of complex disease gene variants by prospective meta-analysis across many epidemiological cohorts, such as in CHARGE and ENGAGE, and in many disease/phenotype focused efforts such as ADSP, IGAP, PERADES, GIANT, GEFOS, REPROGEN, TREATOA, DIAGRAM, etc. Since 2005 the genome wide association study (GWAS) has changed the field of complex genetics, and identified a still growing list of thousands of common genetic variants contributing to disease risk. While this large scale global collaboration has originated from the GWAS era, similar consortia have been built around the genomics datasets with RNA expression profiles, DNA methylation profiles, and the NGS datasets on DNA, RNA and microbiomes, including the BBMRI-NL sponsored BIOS consortium and several CHARGE working groups.

The Rotterdam Study has GWAS data for almost the complete dataset summing to ~ 12,000 DNA samples, and is involved as a major collaborative center for meta-analysis studies of GWAS data, including national programs (BBMRI-exome chip, BBMRI-BIOS), and international consortia (see above). Especially, from the CHARGE consortium many important publications have emerged on a wide variety of phenotypes and diseases from all major research lines in the Rotterdam Study. They are discussed under the subheadings of each individual research line.

### Data collection, storage and management

In the RS-III round, the collection of faeces material has been initiated for the intestinal microbiome analysis. For this a collection pot is distributed at the research center visit which is to be used at home and then returned by postal mail to Erasmus MC where DNA is isolated and stored at − 80 °C. This has been done for ~ 2000 samples in RS-III, and is now continuing for the whole RS study population (with the modification that participants bring their sample directly to the research center to be stored at − 80 °C) following the cycles of visits to the research center, including longitudinal visits.

#### Metabolomics

Two datasets have been created in the Rotterdam Study sub-cohorts that contain information on metabolomics in blood serum or plasma of participants.

- A. As part of the COMBI-BIO consortium, we used large-scale untargeted serum metabolic profiling by proton (1H) nuclear magnetic resonance (NMR) spectroscopy and UPLC Mass Spectrometry to characterize the metabolic signature of 1826 individuals from RS-I-3 in relation with vascular health and cardiovascular disease.
- B. High-throughput metabolomics measurements as a part of the Biobanking and BioMolecular resources Research Infrastructure The Netherlands (BBMRI-NL) initiative have been performed using plasma samples which were collected in EDTA coated tubes. Fasting samples from RS-I (n = 2880), RSII (n = 663), and RS-III (n = 1838) cohorts have been specifically selected in order to maximize the analytical number of prospective gene expression and gut microbiome research in relation to metabolomics. The plasma samples analyzed by the biomarker platform of Nightingale Health using proton nuclear magnetic resonance (NMR) technique. Spectra have been obtained from 600 to 500 MHz instruments, using three molecular windows, namely lipoproteins, lipids and low molecular weight compounds. The spectra were then de-convoluted by Nightingale’s proprietary bioinformatics software leading to quantification of absolute concentrations. The yielding biomarker data contains 228 measurements on apolipoproteins, lipoproteins sub-classes, amino acids, albumin, glucose, glycolysis metabolites, ketone bodies, glycoprotein, sphingolipid, phosphoglyceride, polyunsaturated fatty acids and cholesterols [375].

#### The Human Genomics facility (HuGe-F)

The Rotterdam Study uses the Human Genotyping Facility, HuGE-F (www.glimdna.nl) for all its genomic studies, and which has been generating all GWAS data for the Rotterdam Study as well as its RNA expression profiles, DNA methylation profiles, and all NGS data including whole exome sequences (WES), RNA sequencing data, and the microbiome 16S ribosomal RNA (rRNA) sequencing data.

#### Genome-wide association studies (GWAS) datasets

The GWAS dataset of ~ 12,000 DNA samples from the Rotterdam Study RS-I, -II-, -III cohorts consists of a) a small dataset of ~ 400 women with 500 K Affymetrix arrays (Nsp250 + Sty250; the so-called “pilot” dataset), and b) a large dataset of ~ 12,000 samples consisting of 550 K (RS-I, II; single + duo array format) and 610 K (RS-III; quattro array format) Illumina array genotypes. In the pilot dataset also other array types have been run such as the Illumina Omniexpress 2.5 array, and the new Illumina GSA array and the Affymetrix PMRA array allowing for comparisons.

The Illumina GWAS genotype datasets of the Rotterdam Study also form the basis to generate so-called “imputed” datasets derived thereof. In this process the genotypes of SNPs which have been genotyped in reference datasets (such as HapMap with ~ 2.5 million SNPs genotyped or HRC with 40 million SNPs), are being estimated for all Rotterdam Study samples using the basis Illumina 500 K SNP dataset configurations in each subject. With the advent of large reference datasets becoming available based on whole genome/exome NGS, imputation activities using the Rotterdam Study (RS) GWAS dataset will remain an active area of development. So far, the RS GWAS datasets have been imputed to HapMap version 2 and 3 (with ~ 2.5 million resulting imputed SNP genotypes obtained for the RS dataset), the 1000 genome (1 KG) dataset version Iv3 and IIIv5 (with ~ 30 and 50 million resulting SNP genotypes, respectively), the Genome of the Netherlands (GoNL), the UK10 k whole genome sequencing dataset, and, more recently, the haplotype reference consortium (HRC) r1.1 dataset (~ 40 million SNPs). Especially the latter imputation uses as a reference up to 64,976 haplotypes allowing also the study of less frequent to rare variants and comprising 40 million SNPs, all with an estimated allele count greater than 5.

#### Candidate gene SNPs and special genomic markers

About 300 SNPs in several candidate genes have been individually measured over the past 15 years, (including genes such as ApoE, VDR, ESR1, fibrinogen, etc.). Additionally, for a subset of RS-I samples telomere length (n ~ 1800) and mitochondrial DNA content (n ~ 500) was measured.

### Next generation sequencing datasets

#### Whole genome sequencing (WGS) dataset

The whole genome sequencing dataset consists of 100 samples in RS-I which were sequenced as part of the Genome of the Netherlands (GoNL) [376], with an average sequencing depth of 6× and with improved phasing because of the trio-design.

#### Whole exome sequencing (WES) datasets

WES NGS data in RS-I is available for 2628 samples as part of the NCHA sponsored project and were generated by the HuGe-F facility on the Illumina HiSeq2000 sequencing machines. The samples for this experiment were selected to constitute a random sample from the RS-I dataset. Through a collaborative grant from the NIH Alzheimer initiative (ADSP) we have obtained an additional ~ 1.20 samples with WES NGS data from RS-I generated at the Broad Institute, Boston, USA, of which 50 overlap with the NCHA WES dataset)so net total samples with WES data is 3778). The Rotterdam Study WES dataset is now also part of the so-called commons dataset of the CHARGE consortium with ~ 16,000 WES samples and 5000 WGS samples.

#### RNA sequencing dataset

BBMRI has sponsored a collaborative effort to create a large-scale data infrastructure to work on integrative omics studies in Dutch Biobanks. For this the Erasmus MC HuGe-F genomics facility has generated RNA sequencing profiles of in total ± 4000 individuals of six Dutch biobanks, including the Rotterdam Study. A total number of 900 RS-samples were RNA-sequenced at a depth of 30 million paired end reads. Together with colleagues at UMCG Groningen and LUMC Leiden, the dataset was QC-ed and annotated RNA-expression profiles were generated, and relations between genetics, transcriptomics, and epigenetic measures have been analyzed (see below) and is freely available for all researchers (http://www.bbmri.nl/on__offer/bios/).

### New developments

#### Incidental findings in WES data

Based on the RS WES dataset and the exome chip dataset we have initiated to look for so-called incidental findings which might be clinically relevant. This is done by determining presence of variants in particular sets of genes such as the list of 57 “actionable” genes as established by the American College of Medical Geneticists (AMCG). This research is ongoing, we have established a working group together with Dr Chris O’Donnell, and this is done in collaboration with several groups such as the Broad Institute (Drs. Eric Minikel, Daniel MacArthur) and University of Cologne (Prof. Hilger Ropers). A first result showed that carriers of supposedly pathogenic mutations in the prion gene did not display an evident disease phenotype [377].

WES data was also used to investigate the association between all-cause mortality and carrier-status of somatic mutations in genes linked to clonal expansion of hematopoietic stem cells. We found that, unlike previous reports in predominantly middle-aged individuals, somatic mutations in genes linked to clonal expansion of hematopoietic stem cells do not compromise the 8- to 10-year survival in the oldest old [378].

#### Integrative genomics

Within the Rotterdam Study subcohorts, epigenetic, transcriptomic and microbiome datasets have been generated. Using this data, context-dependent expression quantitative trait loci (eQTL) were identified [379]. In addition, it was found that disease associated genetic variants (GWAS hits) alter transcription factor levels and methylation of their binding sites, offering true biological insight into mechanisms behind the associated GWAS hits [380].

The epigenetic and transcriptomic data have increasingly been explored for associations with disease and traits, and especially environmental factors. Unlike previous efforts in using transcriptomic datasets, this is now also done in large collaborative efforts, increasing robustness and value of the results. Methylation signatures were identified for smoking [62, 381], alcohol consumption [382], low grade inflammation [383], liver enzymes and hepatic steatosis [384], lipids [106], body mass and the adverse outcomes of adiposity [385].

Similarly, transcriptomic profiles were identified for smoking [386], fasting glucose and insulin levels [387] and muscle strength [210]. The first epigenome-wide study was also attempted in relation to bone mineral density variation [388].

A number of studies have focused on the relationship between diverse molecular layers and (biological) aging. A large gene expression meta-analysis in 14,983 individuals identify 1497 genes that are differentially expressed with chronological age. The gene expression profiles were used to calculate the ‘transcriptomic age’ of an individual; differences between transcriptomic age and chronological age were associated with biological features linked to ageing [389]. In a meta-analysis of 3089 individuals were methylation levels were used as a biomarker for “biological age”, often referred to as “epigenetic age”, it was shown that epigenetic age predicts all-cause mortality above and beyond chronological age and traditional risk factors [390].

Furthermore, we showed that blood RNA expression profiles undergo major changes during the seventh decade of life [391]. It was shown to be feasible to accurately estimate human age from blood using information from different molecular layers [392].

#### Microbiome 16S NGS dataset

HuGe-F has optimized and applied stool/faeces collection protocols and used 16S sequencing protocols (NGS of the 16S rRNA v3/v4 area) to characterize the gut/intestinal microbiome. We have collected ~ 2000 stool samples in the RS-III sub-cohort from which DNA has been isolated and which have been sequenced on 16S v3/v4 by NGS on Illumina MiSeq sequencing machines. For other sources of microbiomes (eye, urine, mouth, skin, etc.) several pilot projects have shown their feasibility while sampling and sequencing protocols were optimized (e.g., for some microbiome body niches other 16S areas need to be sequenced). For all these other body niches larger sampling efforts are now ongoing in the ongoing collection rounds of the Rotterdam Study. These can be found under the description of the respective research lines.

## Reproductive traits

### Objective

The main objective of this program is to study frequency and etiology of major disorders of the reproductive system and their risk factors, including age-at-menopause and fertility. Since most analyses involve women, this program is centered around the study of women’s reproductive health. The evaluation of risk factors includes serum measurements of hormones as well as genetic and genomic determinants of reproductive health and related diseases, and studies of the sex chromosomes X and Y. In addition, consequences of these conditions are studied in relation to other aging-related diseases, including cardiovascular disease and disorders of the locomotor system.

### Major GWAS findings

Much of the work of this research is made possible by large-scale collaboration in consortia, some of which focus on one particular disease or trait while others are more broad spectrum strategic collaborations. We are part of several such large consortia studying genetic and epidemiological risk factors for reproductive traits such as CHARGE, REPROGEN, SSCAG and PCOSGEN.

Most attention so far has gone to the study of age-at natural-menopause (ANM) and age-at-menarche in women for which our group was the first to report the major loci for age-at-menopause [393, 394]. Many of these signals were also observed for women of other ancestries [395] although the studies of other ethnicities are smaller and thus lack in power. In the most recent and largest meta-analysis of GWAS of age-at-menopause so far [396], 44 loci were identified among 70,000 women, of which two with rare variants with large effect size (HELB and SLCO4A1) as discovered by exome-array-based meta-analysis. Together, the genome-wide significant variants explain ~ 6% of the genetic variation which went up to 21% if we take all SNPs with *P* < 0.05.

In Mendelian Randomization studies a causal effect was established for age at natural menopause as a risk factor for breast cancer (but not prostate cancer in men), while the effect size was greater for ER-positive than ER-negative breast cancers [396, 397]. Similar MR studies are now ongoing for other common diseases influenced by age-at menopause such as cardiovascular disease and osteoporosis.

Interestingly, the majority of the loci determining age-at-natural menopause involve genes which are important in the DNA damage response and DNA repair pathways which points to the importance of this system in maintaining an error-free stem cell lineage which produce the oocyte. As such the phenotype of age-at-menopause, represents an interesting model for age-related changes in cell function maintenance and functions as a model to identify molecular mechanisms for damage accumulation and repair during ageing [398].

Several diseases related to infertility, such as Early menopause (EM)/Primary Ovarian Insufficiency (POI) and PolyCystic Ovary Syndrome (PCOS) are now subjected to GWAS and look ups with ANM SNPs. In a GWAS of 3493 EM cases and 13 598 controls from 10 independent studies [399], no novel genetic variants were discovered, but the 17 variants previously associated with normal age at natural menopause as a quantitative trait were also associated with EM and primary ovarian insufficiency (POI). In a GWAS of PCOS in 5184 self-reported cases and 82,759 controls [400], 6 loci were identified in/near genes ERBB4/HER4, YAP1, THADA, FSHB, RAD50 and KRR1. MR analyses in this study identified causal roles in PCOS aetiology for higher BMI, higher insulin resistance, later menopause, and lower serum SHBG.

For several endocrine biomarkers GWAS have been performed to identify the genetic loci influencing their serum levels, i.e., testosteron [401], SHBG [402], DHEAS [403], and these are also involved in several MR analyses in relation to major disease endpoints for which these biomarkers have been suggested to be predictive.

In a collaboration with the SSCAG consortium, a recent GWAS of human fertility characteristics (defined as age at first new born (AFB) and number of children ever born (NEB)) in both sexes including 251,151 individuals for AFB and 343,072 individuals for NEB, identified 12 loci [404]. While none of the AFB- or NEB-associated SNPs are associated with age at menopause, there was some overlap with SNPs for behavioral and reproductive phenotypes (such as educational attainment, age-at-menarche, bmi, and age at first sexual intercourse).

### Methods update

Several specific biomarker assessments in ~ 10,000 blood/serum/plasma and urine samples have been done for the diagnosis and evaluation of risk factors of reproductive traits (e.g., steroid hormones; see under “genomics, biomarkers, and microbiome”). Current work involves analyses of X and Y chromosome mosaicisms as can be detected in genomic DNA extracted from blood, and how these mosaicisms change with ageing. In addition, DNA methylation is analyzed as well as microbiome profiles in relation to reproductive traits. The CHARGE-S WES dataset is currently being analyzed for the contribution of rare variants to ANM, while a very large meta-analyses of age-at-menopause is underway involving many more HRC imputed GWAS datasets as well as the UK Biobank dataset of ~ 500,000 samples.

For additional EJE references see [405–415].

## Pharmacoepidemiology

### Objectives

Especially during the past 10 years, there has been a strong increase in the number of automated healthcare databases for pharmacoepidemiology. As most of these databases have limitations because their composition is not only healthcare-driven but may also differ between health insurance systems, they are vulnerable to potential selection and information bias. This clarifies the need for prospectively gathered and standardized information on drugs and disease. In the Rotterdam Study, the role of drugs is studied as determinant of diseases in middle-aged and older community-dwelling individuals. This includes studying efficacy and effectiveness of drugs, as well as adverse reactions to drugs. As the drugs used in the Rotterdam Study are licensed and often on the market since several years, research focuses on determinants which modify the safety and effectiveness of widely used drugs because these often have a great impact on healthcare. The Rotterdam Study is a unique resource for pharmacoepidemiology because of its long follow-up since 1990, complete coverage of more than five million dispensings of prescription-only drugs via 7 automated community-based pharmacies in the region, and repeated interview data for studying drug adherence and ‘over-the-counter’ drugs. In combination with the very rich medical and biological information from repeated interviews and physical, laboratory, imaging data, and genetic and epigenetic determinants, it facilitates a type of pharmacoepidemiologic research which investigates biological-pharmacological mechanisms of drug response.

### Major findings

Below, we summarize findings over the most recent period. Different research themes prevailed, centering around two topics, i.e. studying important drug safety problems and gene-drug interactions of established pharmacologic drug effects. As for the first topic, an important problem for drug licensing authrorities since several years is drug-induced sudden cardiac death. In a recent analysis with data from the Rotterdam Study, we demonstrated that the incidence of sudden cardiac death during the period 1990–2010 declined [416]. Possibly, this is related to the increasing attention for the treatment of cardiovascular morbidity [secondary prevention] and of cardiovascular risk factors such as hypertension and diabetes mellitus [primary prevention]. One of the well-known risk factors for sudden cardiac death is QTc-interval prolongation on echocardiograms [ECGs]. This QTc-interval prolongation is under the influence of genetic variation [417]. An important gene—ABCB1—encodes for the transport protein P-glycoprotein which is abundant in the gut and blood brain barrier. Users of digoxin with a certain variation of the ABCB1-gen had a higher chance of sudden cardiac death [418]. There are many drugs which are able to prolong the QTc-interval, such as serotonin reuptake inhibitors [419]. These SSRI antidepressants are considered to be safer than the traditional tricyclic antidepressants [TCA] when treating elderly with depression but sometimes less effective. However, SSRI are associated with an increased risk of cerebral microbleeds [420]. On the other hand, we found that they are associated with a lower risk of myocardial infarction [421]. Although a large number of studies have been conducted aiming to identify genetic variants associated with antidepressant drug response in depression, only a few variants have been repeatedly identified [422]. Depression is the main indication for antidepressant treatment but results from one of our studies confirmed that antidepressants are also used for off-label indications, subthreshold disorders and complex situations, which were all associated with clinically-relevant depressive symptoms in the middle-aged and elderly population [423]. SSRI use was associated with better subjective sleep, after adjustment for depressive symptoms and concurrent psycholeptic drug use. This suggests that, in clinical practice in the middle-aged and elderly population, the sleep quality of some persons may benefit from, continued, SSRI use [424]. The stronger adverse effect of TCA on the QTc-interval proved to be predominantly related to their more powerful anticholinergic activity. This influence on the autonomic nervous system is associated with an increased heart rate. The consequent decrease of the RR-interval mathematically leads to a prolongation of the QTc-interval according to Bazett without changing the QT-interval itself. Therefore, we demonstrated that the Fridericia-correction leads to a more meaningful measure than the Bazett-corrected one when calculating the QTc-interval from ECGs [425]. We conducted race/ethnic-specific genome-wide interaction analyses of TCAs and resting RR and QT intervals in cohorts of European, African, and Hispanic/Latino (n = 13 808; n = 147 TCA users) ancestry, adjusted for clinical covariates. Among Europeans, TCA interactions with variants in BRE and UBE2E2 were identified in relation to RR intervals. Among Hispanic/Latinos, variants in TGFBR3 modified the relation between TCAs and QT intervals [426]. At variance with that which is suggested in product labelling information, concurrent use of two or more QTc-interval prolonging drugs did not further lengthen the interval to a substantial extent [427]. However, It is clear that the association between QTc and sudden cardiac death is not one-to-one and that other risk factors are important. The role of a decreased serum level of magnesium in cardiac arrhythmias is unclear at the moment but we demonstrated that it was associated with an increased risk of sudden cardiac death [428]. Although hypomagnesemia is uncommon in a situation of normal food intake, longterm use of proton pump inhibitors—for instance indicated in elderly who are also chronic users of NSAIDs—can cause this electrolyte disturbance [429].

We found that SSRI with a high receptor affinity had relatively high serum levels of LDL cholesterol [430]. In another analysis in the Rotterdam Study, we demonstrated that use of SSRI was associated with a stronger weight increase [431]. Also, SSRI decreased insulin secretion in older adults and increased the risk of insulin dependence in patents with type 2 diabetes [432]. In a methodological study we tried to find support for the hypothesis that genome-wide association studies would be able to find genetic determinants for response to SSRI, notably the genes FSHR, HMGB4, PLCB1 and HTR2A [433].

Miscellaneous studies consisted, among others, of risk factors in elderly for resistance to ciprofloxacin in community-acquired urinary tract infections due to E coli. Ciprofloxacin resistance in community-acquired UTI was associated with a high intake of pork and chicken and with concomitant prescription of calcium supplements and proton pump inhibitors [434]. In another study, a nested case–control analysis was performed in which we found that participants with a bacterial gastroenteritis were more likely than controls to be current users of PPIs [435]. Furthermore, In a study in elderly from the Rotterdam Study, B-proof, and LASA cohort, we were able to demonstrate that two variants in cytochrome P450 2C9 modified the fall risk of ageing benzodiazepine users [436].

### Future developments

More and more, pharmacoepidemiology in the Rotterdam Study will concentrate on pharmacological-biological mechanisms of a couple of commonly used benchmark drugs with the help of genetic- and epigenetic techniques, as well as proteomics and metabolomics. Several meta-analyses were performed in recent years. First, in a large international genome-wide association studie of drug-gene interaction, no markers were found for the effect of antihypertensives on cardiovascular disease [437]. One of these antihypertensives, i.e. ACE-inhibitors, is associated with angioedema or coughing which may lead to discontinuation or switching to another antihypertensive. In a second GWAs of 972 switchers from ACE-inhibitors, eight SNPs within four genes reached the genome-wide association study significance level in the meta-analysis: RNA binding protein, Fox-1 homolog (Caenorhabditis elegans), γ-aminobutyric acid receptor subunit γ-2, sarcoma (Src) homology 2 (SH2) B adaptor protein 1 and membrane bound O-acyltransferase domain containing 1 [438].

Third, in a large-scale GWAs of the effect of sulfonylurea hypoglycemics on QT, JT, and QRS intervals in 11 ethnically diverse cohorts that included 71 857 European, African-American and Hispanic/Latino ancestry individuals eight novel pharmacogenomic loci met the threshold for genome-wide significance. A pharmacokinetic variant in CYP2C9 (rs1057910) that has been associated with sulfonylurea-related treatment effects and other adverse drug reactions in previous studies was replicated [439]. Fourth, we performed a large-scale meta-analysis across the cohorts of the Metformin Genetics Consortium (MetGen). Nine candidate polymorphisms in five transporter genes (organic cation transporter [OCT]1, OCT2, multidrug and toxin extrusion transporter [MATE]1, MATE2-K, and OCTN1) were analyzed in up to 7968 individuals. None of the variants showed a significant effect on metformin response in the primary analysis, or in the exploratory secondary analyses, when patients were stratified according to possible confounding genotypes or prescribed daily dose of metformin [440]. However, The C allele of rs8192675 in the intron of SLC2A2, which encodes the facilitated glucose transporter GLUT2, was associated with a 0.17% greater metformin-induced reduction in hemoglobin A1c (HbA1c) in 10,577 participants of European ancestry. rs8192675 was the top cis expression quantitative trait locus (cis-eQTL) for SLC2A2 in 1226 human liver samples, suggesting a key role for hepatic GLUT2 in regulation of metformin action [441]. Fifth, we performed a meta-analysis of genome-wide association studies (GWAS) to identify variants with an effect on statin-induced high density lipoprotein cholesterol (HDL-C) changes. The 123 most promising signals were followed up in an independent group of 10 951 statin-treated individuals, providing a total sample size of 27,720 individuals. The only associations of genome-wide significance were between minor alleles at the CETP locus and greater HDL-C response to statin treatment [442].

For additional EJE references see [435, 443–454].

## Imaging studies

The Population Imaging Unit within the Rotterdam Study aims to assess (quantitative) imaging biomarkers of disease in a pre-symptomatic phase at the population level [455] Advantages of imaging measures include that they mark early disease, can be assessed reliably and reproducibly, and are quantitative rather than qualitative which makes them more powerful than most conventional outcome measures such as clinical phenotypes.

The main imaging modalities that are currently being applied in the Population Imaging Unit are multidetector computed tomography (MDCT) and magnetic resonance imaging (MRI). The imaging infrastructure has been described extensively in the previous study design papers [6, 19].

Important updates on our research since our last report are the following:

### Incidental findings on imaging

We previously indicated that assessment and management of incidental findings is of great importance in large-scale imaging studies like ours. Unfortunately, guidelines are lacking and information on natural course is still scarce. We have tried to close these gaps by describing an ethical framework which can be used in designing studies [456, 457], and we have reported the natural course and clinical management of findings in our study since 2005 [458]

### Imaging of age-related brain changes and neurological diseases

An important focus in our work is on quantitative markers that signify preclinical change, preferably in the earliest state of disease. In this context, we have explored in recent years how structural connectivity in the brain changes with age [459,] and also how these changes affect cognition [460]. Also, we showed that worse microstructural integrity related to higher mortality [461]. Furthermore, we found that future stroke is predicted not only by prevalent vascular lesions (such as infarcts or white matter hyperintensities) but also by subtle alterations in the microstructure of normal-appearing white matter [254]. Inclusion of this effect in risk prediction models produced a significant advantage in stroke prediction compared with the existing Framingham Stroke Risk Profile.

After introduction of resting-state functional MRI, we have explored how (change in) brain structure drive brain function, and found that white matter pathology can decrease tract-specific functional connectivity, both in direct and indirect connections [462]. These results provide further evidence for the so-called “connectivity hypothesis”. We are currently extending this work by defining the “disconnectome” in the brain, and by studying how functional brain connectivity changes with age and affects cognitive functioning.

Despite increased understanding of microbleed pathology, their clinical implications remained largely unknown. We studied microbleeds as a determinant of stroke and dementia and found that microbleeds associated with an increased risk of recurrent and first-ever stroke, both ischemic and hemorrhagic [463]. Our results confirm that the increased risk is not confined to people with prior strokes, and can be extrapolated to people from the general population. Another finding was the correlation in anatomical location between cerebral microbleeds and intracerebral haemorrhage [463]. Finally, in longitudinal studies we found that microbleed presence related to decrease in cognitive functioning and an increased the risk of dementia, including Alzheimer’s dementia [464]. Taken together, our findings suggest that cerebral microbleeds may represent an imaging marker of active vasculopathy, which serves as a predictor of both ischemic and hemorrhagic brain lesions and neurodegeneration.

### Imaging of atherosclerosis and cardiovascular diseases

As described previously [6], we make use of both MDCT and MRI to image atherosclerotic calcifications (in multiple vessel beds), plaque burden and atherosclerotic plaque composition (in the carotid). Important new reports describe the determinants of overall plaque burden and how plaque composition relates to a history of stroke [465, 466]. In recent years, we have expanded our interest in imaging markers of cardiovascular disease towards epicardial fat [467, 468] and aortic valve calcification [253, 469].

In a preliminary investigation, we have applied computational fluid modelling to investigate the relation between shear stress and vulnerable plaque components, and found that higher shear stress related to intraplaque haemorrhage [470]. We are currently expanding this study to measure shear stress in over 2000 carotid MRI scans from our population. Using serial imaging, we were able to describe determinants of change in plaque components over time [471].

### Future developments

As also mentioned above, focus has shifted in recent years from purely structural imaging to also including functional imaging data, by incorporating resting-state functional MRI into the brain imaging protocol. Changes in the intrinsic activity of resting-state networks are presumed to represent alterations in functional brain connectivity and may mark neurodegeneration in an early, presymptomatic stage. We will further explore the value of functional imaging as an early imaging marker for dementia, by itself or in combination with other imaging markers and risk factors.

Another development that has set in and will continue in the coming years is that we do not regard the brain as a stand-alone organ, but rather view it in the context of the rest of the body and other diseases outside the brain. In the past years, we have found abundant evidence that pathology in the brain is linked to (sub)clinical pathology elsewhere in the body [314, 315, 472–474], and we will explore these interconnections further.

Finally, an emerging potential marker is Virchow–Robin (VR) spaces, or enlarged perivascular spaces, spaces filled with interstitial fluid that surround the blood vessels in the brain and which can be dilated. Despite increasing literature on these dilated VR spaces, a major limitation of current research is the lack of a robust and generalizable rating method on MRI. After successful implementation of a new rating method, we are currently investigating the value of VR spaces in a large consortium of other population-based studies [475] (www.uconsortium.org).

Besides ever-increasing advances in imaging hardware, software and sequence design, major advances in the short and long run are to be expected from (fully) automated image analysis. Computer processing of images will enable to make fully use of all information contained within the image, introducing new imaging biomarkers. Besides, the vast amount of imaging data that are acquired in population-based studies like the Rotterdam Study renders visual assessment or manual measurements virtually impossible, strengthening the need for (fully) automated methods of data extraction and analysis.

For additional EJE references see [457, 476].

## Otorhinolaryngology

### Objectives

Otolaryngological research in the Rotterdam Study focuses on the frequency, etiology and consequences of hearing loss. Age-related hearing loss is a common disorder that deprives older people of key sensory input. It leads to social withdrawal and is even been found to be independently associated with poorer cognitive functioning and incident dementia. Still, little is known about the mechanisms that are responsible for developing hearing loss and the way it affects general cognitive functions within the elderly population. Determinants of interest are genetic factors, cardiovascular disease, use of medication, endocrine diseases and neuro-epidemiological factors.

### Methods

Hearing loss is assessed at both ears by performing pure-tone audiometry in a sound proof room. Hearing thresholds are determined with headphones at frequencies 0.25, 0.5, 1, 2, 4 and 8 kHz. To distinguish between cochlear and middle-ear pathology, also bone-conduction thresholds are measured at frequencies 0.5 and 4 kHz. Additionally, speech perception in noise is tested at the better ear, using a validated triplet digit test [477] with speech-shaped noise at a fixed presentation level of 65 dB SPL. The ability to understand speech in noise is a functional measure that includes both sensory and central aspects of the auditory system.

From a subset of the participants peripheral vestibular function is assessed by The Head Impulse Test (HIT), which measures the vestibule-ocular reflex (VOR) for a number of sudden head movements initiated by the tester [478]. Gain and delay are the main parameters that will be used to quantify vestibular function. The main goal is to analyze possible associations between cochlear and vestibular dysfunction, as both sensory organs are connected and use similar mechanisms.

The general interview contains ten questions related to hearing and balance problems. In case of hearing-aid use, the participant has to answer five additional questions of the International Outcome Inventory of Hearing Aids (IOI-HA) [479]. In case of frequent tinnitus, ten additional questions of the Short Tinnitus Handicap Inventory (THI-S) are added [480].

### Major findings

As expected, we found a high prevalence of hearing loss in population of the Rotterdam Study [481]. In the population of 65 years and older, 30% had a hearing loss of 35 dB HL of more. However, the difference in hearing between sexes was considerably less than previously reported. This is probably due to changing lifestyle and environmental circumstances. A general association study including relevant determinants revealed that hearing loss was independently associated with age, education, systolic blood pressure, diabetes mellitus, BMI, smoking and alcohol consumption [482]. Remarkably, different associations were found for low- and high-frequency loss, as well as between men and women, suggesting that different mechanisms are involved in the etiology of age-related hearing loss. Furthermore, a strong and consistent relation was found between hearing loss and a decreased ability to understand speech in noise [483], which confirms the substantial impact of hearing loss on social interaction. To further analyse the possible impact of hearing on general functioning, we studied the relation of hearing loss with brain-related parameters. This study revealed that hearing loss was independently associated with a smaller brain volume [484], which was mainly driven by a smaller white matter volume throughout the brain in case of poorer hearing. Genetic susceptibility to age-related hearing loss is another important topic that is being analysed at the moment in a large meta-analysis of the international CHARGE consortium.

For additional EJE references see [485–487].

## Management

The Rotterdam Study is directed by a Management Team comprising the scientific principal investigators Sarwa Darwish Murad (PI Hepatic diseases), Cornelia van Duijn (PI Genetic epidemiologic studies), Oscar Franco (PI Cardiovascular diseases and ErasmusAGE), André Goedegebure (Otolaryngological diseases), Albert Hofman (Epidemiology), Arfan Ikram (chairman, PI Rotterdam Study), Caroline Klaver (PI Ophthalmic diseases), Tamar Nijsten (PI Dermatological diseases), Robin Peeters (PI Internal Medicine), Bruno Stricker (PI Pharmaco-epidemiology), Henning Tiemeier (PI Psychiatric diseases), André Uitterlinden (PI Genomic studies), and Meike Vernooij (PI Population Imaging); and Jan Heeringa, MD, PhD, study coordinator, Eric Neeleman, head IT, and Frank van Rooij, MSc, head data-management.

## Emeritus principal investigators

The following persons are Principal Investigator Emeritus of the Rotterdam Study:

Frank van den Ouweland (PI Internal Medicine 1990–1992), Diederick Grobbee (PI Cardiovascular diseases 1990–1996), Albert Hofman (PI Neurological diseases 1990–1996), Paulus de Jong (PI Ophthalmic diseases 1990–2005), Huibert Pols (PI Internal Medicine 1993–2006), Monique Breteler (PI Neurological diseases 1997–2010), Gabriel Krestin (PI Population Imaging 1998–2010), Johannes Vingerling (PI Ophthalmic diseases 2005–2010), Jacqueline Witteman (PI Cardiovascular diseases 1997–2011), Ernst Kuipers (PI Internal Medicine 2007–2013), Harry Janssen (PI Hepatic diseases 2007–2013).
